# Assessment of Phthalate Esters and Physiological Biomarkers in Bottlenose Dolphins (*Tursiops truncatus*) and Killer Whales (*Orcinus orca*)

**DOI:** 10.3390/ani14101488

**Published:** 2024-05-17

**Authors:** Leila S. Lemos, Amanda C. Di Perna, Karen J. Steinman, Todd R. Robeck, Natalia S. Quinete

**Affiliations:** 1 Institute of Environment, Florida International University, North Miami, FL 33181, USA; 2 Emerging Contaminants of Concern Research Laboratory, Department of Chemistry & Biochemistry, College of Arts, Sciences, and Education, Florida International University, North Miami, FL 33181, USA; adipe001@fiu.edu; 3 SeaWorld & Busch Gardens Species Preservation Laboratory, United Parks and Resorts, San Diego, CA 92109, USA; karen.steinman@unitedparks.com (K.J.S.); todd.robeck@unitedparks.com (T.R.R.); 4 United Parks and Resorts, 7007 Sea Harbor Drive, Orlando, FL 32821, USA

**Keywords:** aldosterone, contaminants, cortisol, corticosterone, glucocorticoids, hydrogen peroxide, lipid peroxidation, oxidative stress, steroid hormones, MDA

## Abstract

**Simple Summary:**

Delphinids are top predators and display long lifespans, making them susceptible to accumulating harmful environmental contaminants within their tissues, which may lead to potential adverse effects on their physiology and overall health. This study aimed to investigate exposure to phthalates in aquarium-based bottlenose dolphins and killer whales and explore potential correlations with individual physiological responses while accounting for sex, age, and reproductive stage. We found that all individuals were exposed to phthalates, including newborn dolphins. Phthalates were also correlated with aldosterone concentrations in killer whales, suggesting that this class of contaminants may exert disruptive effects on the endocrine system and metabolism of dolphins. This study could help us better understand the physiological mechanisms and effects of phthalate exposure in delphinids, which could lead to stricter contaminant discharge regulations, improving both human and aquatic/marine health.

**Abstract:**

There is growing concern about the potential adverse health effects of phthalates (PAEs) on human health and the environment due to their extensive use as plasticizers and additives in commercial and consumer products. In this study, we assessed PAE concentrations in serum samples from aquarium-based delphinids (*Tursiops truncatus*, n = 36; *Orcinus orca*, n = 42) from California, Florida, and Texas, USA. To better understand the physiological effects of phthalates on delphinids, we also explored potential correlations between phthalates and the biomarkers aldosterone, cortisol, corticosterone, hydrogen peroxide, and malondialdehyde while accounting for sex, age, and reproductive stage. All PAEs were detected in at least one of the individuals. ΣPAE ranges were 5.995–2743 ng·mL^−1^ in bottlenose dolphins and 5.372–88,675 ng·mL^−1^ in killer whales. Both species displayed higher mean concentrations of DEP and DEHP. PAEs were detected in newborn delphinids, indicating transference via placenta and/or lactation. Linear mixed model results indicated significant correlations between aldosterone, month, location, status, and ΣPAEs in killer whales, suggesting that aldosterone concentrations are likely affected by the cumulative effects of these variables. This study expands on the knowledge of delphinid physiological responses to PAEs and may influence management and conservation decisions on contamination discharge regulations near these species.

## 1. Introduction

Phthalate esters (PAEs) are a group of synthetic chemicals widely used in the production of plasticizers and additives in consumer products, including personal care products, cosmetics, pharmaceuticals, food packing, detergents, and children’s toys [1]. There are growing concerns about the potential adverse effects of PAEs on both human health and the environment, given their status as one of the most common human-contact chemical classes [2]. Previous studies have associated PAEs with several health issues in humans, including diabetes; insulin resistance; weight and obesity; allergy and asthma; developmental and reproductive anomalies; and impacts on hepatic and renal function, thyroid signaling, immune function, and metabolism [3,4,5,6,7,8,9,10,11,12,13]. It has also been proven that PAEs induce oxidative stress and cell degenerative processes by increasing intracellular reactive species [14]. PAEs are also considered endocrine disruptors and have been associated with shifts in hormone concentrations, including reduced testosterone [15], elevated progesterone [16,17], reduced cortisol and corticosterone [18,19], reduced aldosterone [20], and reduced triiodothyronine (T3) and free thyroxine (T4) [21]. Moreover, certain PAEs (i.e., DEHP—Di(2-ethylhexyl) phthalate; and BBP—Benzylbutyl phthalate) have been classified as potential carcinogens for humans by the United States Environmental Protection Agency (U.S. EPA) [22]. Although the health effects of PAEs on humans have been sufficiently examined, comprehensive evaluations regarding wildlife exposure to phthalates and their subsequent impacts are still lacking [23].

Monitoring PAE exposure in bioindicator species can be a valuable tool to evaluate the influence of these emerging organic contaminants on a specific species and its surrounding environment, with the potential to identify critical contaminated areas and potential sources of pollution [24,25,26]. In the marine environment, marine mammals have been widely utilized as efficient indicators of environmental chemical pollution and its consequences due to their higher position in the food chain and long lifespan [27,28,29]. Prior research has found exposure to different phthalate compounds in varied cetacean species, including bottlenose dolphins (*Tursiops truncatus* [30,31,32,33,34,35,36,37]), killer whales (*Orcinus orca* [38]), sperm whales (*Physeter macrocephalus* [38]), long-finned pilot whales (*Globicephala melas* [38]), short-finned pilot whales (*Globicephala macrorhynchus* [30]), white-beaked dolphins (*Lagenorhynchus albirostris* [38]), harbour porpoises (*Phocoena phocoena* [38]), Risso’s dolphins (*Grampus griseus* [30,31]), striped dolphins (*Stenella coeruleoalba* [30,31]), pygmy sperm whales (*Kogia berviceps* [30]), Fraser’s dolphins (*Lagenodelphis hosei* [30]), and fin whales (*Balaenoptera physalus* [31]).

However, there is a noticeable gap in the literature concerning studies on the impact and health risks of phthalate exposure to marine mammals. After a thorough review, we were only able to identify one study that has attempted to correlate phthalate exposure with physiological responses in cetaceans. In this study, Dziobak et al. (2022) have described a positive relationship between blood thyroid hormone concentrations (free thyroxine) and urinary MEHP (mono(2-ethylhexyl) phthalate), a DEHP metabolite, for both adult female and male dolphins. They suggest that DEHP exposure may be impacting thyroid hormone homeostasis.

Various stress biomarkers can be used as tools to identify potential physiological responses to one or more stressors, including hormones and oxidative stress biomarkers. Steroid hormones such as the glucocorticoids (GCs) cortisol and corticosterone, and the mineralocorticoid aldosterone regulate metabolic, immune, and stress responses [39,40]. GCs coordinate adaptive responses during demanding situations by triggering a cascade of effects like energy mobilization and increases in heart and respiratory rates [39,40], while aldosterone is essential for regulating salt and water balance, leading to water retention and subsequent increases in blood volume and pressure [41,42]. During stress responses, both GC and aldosterone secretion are stimulated [40,43], restoring ionic and osmotic balances, stabilizing blood pressure and supporting cardiovascular function [44]. Concurrently, oxidative stress biomarkers such as hydrogen peroxide (HP; H_2_O_2;_ type of reactive oxygen species—ROS) and malondialdehyde (MDA; lipid peroxidation metric) serve as indicators of oxidative damage. HP is a byproduct of normal cellular metabolism, such as aerobic respiration or inflammatory processes; and other factors such as excessive exposure to UV radiation, long-term stress conditions (such as exposure to contaminants), intense physical exercise, and improper diet [45]. Although cells have antioxidant defense mechanisms, excessive ROS can cause an imbalance, leading to oxidative stress. This stress is exemplified when ROS target polyunsaturated fatty acids in cell membranes, initiating a chain reaction known as lipid peroxidation and causing cell damage through byproducts like MDA [46].

It is unclear how PAEs affect cetaceans physiologically. There are no studies available associating PAEs with the stress biomarkers proposed in the present study. Nevertheless, existing research in other mammalian species provides substantiated evidence that PAEs may exert effects on physiological parameters such as aldosterone, cortisol, corticosterone, HP, and MDA. Therefore, we hypothesize that similar physiological mechanisms and effects may occur in cetaceans, and exploring these potential correlations will likely bring valuable insights to our overall understanding. Bottlenose dolphins (*Tursiops truncatus*) and killer whales (*Orcinus orca*), like other cetaceans, are top predators and have a long lifespan, making them susceptible to accumulating harmful environmental contaminants such as PAEs. Therefore, the objectives of this study were to analyze available historic serum samples from bottlenose dolphins and killer whales housed in the SeaWorld aquariums within the United States to (1) determine PAEs profiles; (2) assess individual physiological responses by quantifying stress-related hormones (aldosterone, cortisol, corticosterone), and oxidative stress biomarkers (hydrogen peroxide and MDA concentrations); (3) investigate the possible occurrence of PAE transference from mother to calf via placenta and lactation; and (4) explore potential correlations between PAE concentrations and physiological responses while accounting for sex, age, reproductive state, location, and time.

## 2. Material and Methods

### 2.1. Study Animals and Sampling

This research was a retrospective study, relying on blood samples collected and banked during previous routine health exams of two aquarium-based delphinid species. Bottlenose dolphins were housed in two SeaWorld Parks in Orlando, FL, and San Diego, CA. Their enclosures consisted of ≥850 m^3^ of either natural/processed (SWC) or manufactured saltwater (SWT, SWO) with water temperatures ranging from 14 to 28 °C [47]. Salinity varied with location and source with SWC having an annual mean of 33.6 + 0.6 ppm, while at SWT and SWO, the annual mean salinities were both 27.6 + 2.2 ppm. Their diet comprised frozen–thawed whole fish purchased from a seafood distributor (McRoberts Sales Co., Inc., Ruskin, FL, USA) and included capelins (*Mallotus villosus*), herrings (*Clupea harengus*), and Columbia River smelts (*Thaleichthys pacificus*). The dolphins received a daily diet equivalent to around 4–5% of their body weight.

Killer whales were housed in three different SeaWorld Parks located in Orlando, FL, San Antonio, TX, and San Diego, CA. Their enclosures comprised a minimum of 19,000 m^3^ of either natural/processed or artificially produced saltwater with a water temperature of around 14 °C throughout the year [48]. Their diet also consisted of frozen–thawed fish purchased by the same distributor as bottlenose dolphins, and included capelins (*Mallotus villosus*), herrings (*Clupea harengus*), sardines (*Sardinops sagax*), Pacific mackerels (*Scomber japonicus*), and pink salmon (*Oncorhynchus gorbuscha*). Killer whales were provided with a daily diet equivalent to around 2–3% of their body weight.

All fish offered to both species underwent grading for human consumption, and the animals were additionally supplemented with Vita-Zu Marine Mammal tablets without Vitamin A (Mazuri, St. Louis, MO, USA).

From 1994 to 2020, 78 blood samples were collected from 27 individuals of bottlenose dolphins (n = 36 samples; 13 individuals) and killer whales (n = 42 samples; 14 individuals) at different reproductive stages (Tables S1 and S2). Samples were obtained from unrestrained animals during routine health exams. Each animal voluntarily presented its ventral fluke surface for the attending veterinarian to perform venipuncture on the central fluke vein, using a 19-gauge, 1.5-inch needle. About 20 cc of whole blood was collected using BD Vacutainers (Becton Dickinson, Franklin Lakes, NJ, USA) containing either K-EDTA or activated thrombin. Within 30 min of blood collection, samples were centrifuged at 1500 rpm for 10 min, the serum was separated, decanted, and then frozen and banked at −80 °C until sample analysis.

### 2.2. Chemicals and Materials

Hexane, methylene chloride (DCM), florisil, and anhydrous sodium sulfate were purchased from Fisher Scientific (Hampton, NH, USA). Three phthalate native standards (dimethyl phthalate—DMP [99%, 250 g], dibutyl phthalate—DBP [99%, 500 g], and diethyl phthalate—DEP [99%, 250 g],) were purchased from Alfa Aesar (Ward Hill, MA, USA). Three other native standards (benzyl butyl phthalate—BBP, Di(2-ethylhexyl) phthalate—DEHP, and dioctyl phthalate—DOP) and six phthalate isotopically mass-labeled standards (BBP-d4, DBP-d4, DEHP-d4, DEP-d4, DMP-d4, and DOP-d4) were purchased from AccuStandards (100 μg·mL^−1^ in methanol; New Haven, CT, USA) and Ultra Scientific (100 μg·mL^−1^ in methanol; North Kingstown, RI, USA). Stock solutions of 100 μg·mL^−1^ of the native standards DMP, DBP, and DEP were prepared in methanol. All six native standards were combined into one solution of 5 μg·mL^−1^ in methanol, and all six mass-labeled standards that were used as surrogates were combined into a second solution of 5 μg·mL^−1^, also in methanol. A solution of tetrachloride-m-xylene (TCMX; 5 μg·mL^−1^) was purchased from Supelco (St. Louis, MO, USA) and prepared in methanol to be used as a recovery internal standard. All stock solutions were stored in the freezer at −8 °C.

RIPA buffer and EDTA (0.5 M, pH 8.0, autoclaved) used in the oxidative stress analyses were purchased from Boston BioProducts (Milford, MA, USA), and potassium phosphate monobasic (Tech grade, 500 g) was purchased from Fisher Scientific (Hampton, NH, USA). Assay kits were purchased from Cayman Chemical (TBARS—kit #10009055; Ann Arbor, MI, USA) and BioAssay Systems (hydrogen peroxide—kit #DIOX-250; Hayward, CA, USA). Hormone analyses utilized commercially available enzyme-linked immunoassay kits (ELISA) purchased from Arbor Assays (Ann Arbor, MI, USA) for corticosterone (kit #K014) and aldosterone (kit #K052). All chemicals and reagents for the commercial kits were provided by the manufacturer. Additionally, the cortisol assay was an “in-house assay”, utilizing antibodies and enzyme conjugates provided by UC Davis (Davis, CA, USA). Other chemicals and reagents used in the cortisol assay included sodium chloride (S5886), Tween 20 (P1379), sodium bicarbonate (S2127), sodium carbonate (S8875), sodium phosphate (monobasic [S9638] and dibasic [S0876]), bovine serum albumin (A7906), citric acid (C0759), 2,2′azino-bis(3-ethylbenzothiazoline-6-sulfonic acid) diammonium salt (A1888), hydrogen peroxide (H1009), and cortisol standard (386698). All these chemicals and reagents were provided by Sigma Aldrich (St. Louis, MO, USA).

### 2.3. PAEs Sample Preparation

All glassware used in the PAEs sample preparation was combusted at 450 °C for 14 h and rinsed twice with hexane and twice with DCM to remove any potential cross-contamination. All solvents used in the procedure were HPLC-grade and were tested for PAEs contamination. Plastic components were not utilized during this analysis. Two hundred microliters of each sample were pipetted into clean labeled amber glass tubes and then spiked with 50 μL of a surrogate mixture (5 ng·mL^−1^). Two mL of DCM were added to the vials and vortexed for two minutes. Tubes were then centrifuged (Thermo Scientific Sorvall ST 8, Waltham, MA, USA) at 2500 rpm for 10 min and the supernatant was transferred to a new vial using a Pasteur pipette (Fisher Scientific, Waltham, MA, USA).

Then, the samples underwent a first clean-up procedure using a funnel–flask system containing glass wool and sodium sulfate to eliminate excess water from the samples. The system was pre-conditioned by adding 10 mL of DCM. Samples were then concentrated to 1–2 mL using a rotavapor (Brinkmann Büchi RE 111, New Castle, DE, USA). Samples underwent a second clean-up procedure to remove any interferents by using a chromatography column containing glass wool, florisil, and sodium sulfate. The system was pre-conditioned by adding 10 mL of DCM. Sample extracts were added to the column using a Pasteur pipette. Each of the vials was rinsed with 1 mL of DCM and transferred into the column. The column was then eluted by adding 9 mL of DCM. Samples were concentrated once again using the rotavapor, until they reached <0.5 mL. Vial walls were rinsed with DCM until they reached a final volume of 0.5 mL. Using glass pipettes, the sample extracts were transferred to GC-MS glass vials. Fifty microliters of a final IS (tetrachloro-m-xylene—TCMX) were added to each of the vials to verify instrument accuracy.

Samples were analyzed by gas chromatography with a single mass spectrometer (GC-MS; Thermo Scientific ISQ 7000 single quadrupole interfaced to a Thermo Scientific Trace 1310 Gas Chromatograph, West Palm Beach, FL, USA). The column used in the analysis was a TG-5SILMS (Thermo Scientific, 30 m × 0.25 mm × 0.25 μm). The GC column/oven conditions (Table S3), MS parameters (Table S4), and summary of the instrumental acquisition method (Table S5) can be found in the Supplementary Information. Six phthalates were selected from the priority list of pollutants from the United States Environmental Protection Agency (USEPA) and the European Union (EU; [49]): BBP, DBP, DEHP, DEP, DMP, and DOP (listed in Table S5). Samples were diluted and re-run if sample concentrations were above the calibration curve range. All samples were processed and analyzed in 2022. Generated raw data were processed using the Chromeleon 7 software (version 7.3). Values below the method detection limit (MDL) were assigned as MDL/2 for statistical purposes.

### 2.4. PAEs Method Validation and Quality Control/Quality (QC/QA)

Method validation assessed linearity, sensitivity, inter- and intra-day precision and accuracy, and matrix effects. We used procedural blanks, spiked procedural blanks, and a spiked matrix per batch of 10 samples. To ensure analytical data quality, a continuing calibration verification (CCV) was also run after every batch. A CCV deviance of no more than 30% was acceptable. In case of a higher deviance, the instrument was cleaned and calibrated, and new injections were performed.

Calibration curves of eight points (5 to 1000 ng·L^−1^) were prepared and run before every set of 30 samples. Linearity was evaluated by plotting the calibration curves using the area ratio against the compound concentration. Curves showed R^2^ coefficients greater than 0.99 for all the compounds (Table S6). Sensitivity was evaluated by estimating the method detection limit (MDL), defined as the lowest point of the calibration curve that could be detected on the instrument (5 ng·mL^−1^; Table S6).

Precision, accuracy, and matrix effect were assessed using bovine plasma. The material was reconstituted in the proportion of 10:1 (sample–LC-MS water). Intra- and inter-day precisions were evaluated by analyzing twenty replicates, nine within the first day, four within the second day, and seven within the third day of analysis. Precision was calculated in terms of relative standard deviation (RSD; Table S6). Intra-day RSD was <27% for all compounds, while inter-day RSD was <23% for all compounds. To evaluate the accuracy of the method, analyte recoveries (%) were calculated by subtracting PAE concentrations found in unspiked samples from spiked samples and dividing by the added PAE concentration (50 mg·L^−1^; Table S6). Most of the compounds displayed recoveries ranging from 60 to 140%. The exceptions were DEHP (72–247%) and DOP (22–145%; Table S6). Mean surrogate recoveries related to the TCMX (internal standard) responses ranged from 39% in DEP to 110% in BBP.

To investigate if matrix effects (MEs) affected our analyses, MEs were calculated using the following formula:

$$MEs\;\left(\%\right):\left(\left(\frac{\left[spiked\;sample\;matrix-unspiked\;sample\;matrix\right]}{spiked\;concentration\;in\;methanol}\right)-1\right)\times 100$$

Low matrix effects were observed for most of the compounds. Higher suppression (−54.4%) and enhancement (94.5%) were noted for DEHP (Table S6). The analytical method applied herein was successfully validated following the US Environmental Protection Agency guidelines [50].

### 2.5. Hormone Analysis

Serum hormone extraction efficiency and use in aldosterone, cortisol, and corticosterone assays have been previously validated and described for bottlenose dolphins [47,51] and killer whales [48,52,53].

Aldosterone concentrations were measured in bottlenose dolphins (n = 27) and killer whales (n = 30) using a commercial EIA kit. Unfortunately, not all samples could be analyzed for aldosterone due to an insufficient sample volume. All assays were performed according to the manufacturer’s protocol [54]. All samples were analyzed in duplicate and any sample with a coefficient of variation (CV) >10% between replicates was repeated. Inter-assay variation for the two controls with antibodies at 30 and 70% binding was 1.6 and 5.6%, respectively (n = 3).

Cortisol concentrations were measured in bottlenose dolphins (n = 36) and killer whales (n = 42) using an “in-house” single antibody direct enzyme immunoassay (EIA), as previously described by Munro and Lasley [55]. All samples were analyzed in duplicate, and any sample with a CV higher than 10% between replicates was re-run. The inter-assay CV for the two quality controls with antibodies at 30 and 70% binding was 6.1% and 11%, respectively (n = 3).

Corticosterone concentrations were measured in bottlenose dolphins (n = 31) and killer whales (n = 42) using a commercial EIA kit. Unfortunately, not all samples could be analyzed for corticosterone due to an insufficient sample volume. All assays were performed according to the manufacturer’s protocol [56]. All samples were analyzed in duplicate, and samples with a CV >10% between replicates were re-run. the inter-assay variation for the two controls with antibodies at 30 and 70% binding was 1.3 and 2.5%, respectively (n = 3).

### 2.6. Oxidative Stress Analysis

All serum samples, standards, and controls were assayed in duplicate and followed the manufacturer’s protocol for the TBARS (measured by MDA; [57]) and Hydrogen Peroxide (HP; [58]) kits. Assays were quantified using a microplate reader (BIO-TEK Synergy HT) at absorbance spectrum ranges of 530–540 nm for TBARS and 540–610 nm for Hydrogen Peroxide. Data analysis was conducted in the Gen5 software version 3.00. The limit of detection (LOD) for the HP assay was 0.2 μM, as determined by the manufacturer [58]. The LOD used for the TBARS assay was 0.625 μM, which is the smallest point on the curve [57]. Samples with concentrations exceeding the calibration curve range were appropriately diluted and reassayed. Samples were also reassayed if the coefficient of variation (CV) between duplicates was >15%.

### 2.7. Statistical Analysis

A Shapiro–Wilk normality test was performed to verify the distribution of the numeric variables. After verifying that most of the variables displayed a non-Gaussian distribution, a log transformation (log-normal [value + 1]) of the values was conducted. Statistical tests were performed in R software version 4.2.3 [59], with alpha set at 0.05.

Mean, standard deviation, minimum and maximum values, and detection frequency were calculated for all PAEs and stress biomarkers. Linear regressions were conducted using the *lm* function in R to verify correlations between PAEs and stress biomarker concentrations. *t*-tests and one-way ANOVA tests were also performed to verify significant variations among two or three grouping factors, respectively. A Tukey’s posthoc test was conducted after a significant one-way ANOVA result to determine which factors differed.

A series of linear mixed models (LMM) for each of the biomarkers were performed in the R software using the *lme4* package [60]. We assessed the influence of the variables dolphin identification (ID), month, year, location, age, demographic status, ΣPAEs, and other stress biomarkers when applicable. We used dolphin ID as a random effect in all models to account for pseudoreplication. Model selection was based on the lowest Akaike’s information criterion AIC [61]. Some models displayed a singular fit warning, which may indicate model overfitting. Models were then limited to a single random effect (dolphin ID) and less fixed effects by excluding correlated variables (i.e., cortisol, corticosterone, and aldosterone), but the warning message continued. Another possibility for this warning message is the low sample size when grouping the data by the different effects [62]. Because this issue would not be solved with the further exclusion of variables, we conducted the analyses based on parsimony and inclusion of relevant variables for each of the stress biomarkers. F-statistics and *p*-values were assessed using the *lmerTest* package in R [63], and model fit was assessed by the marginal R_2_ (R_2_m: variance explained by fixed effects) and the conditional R_2_ (R_2_c: variance explained by both fixed and random effects) using the *MuMIn* package [64,65].

## 3. Results and Discussion

### 3.1. Delphinid Populations Description

Seventy-eight samples of 13 individuals of bottlenose dolphins (9 females and 4 males; n = 36) and 14 individuals of killer whales (9 females and 5 males; n = 42) were collected at varied temporal intervals and reproductive stages (Tables S1 and S2). Four mother–calf pairs, two pairs from each species, were also sampled to investigate contaminant transference via placenta and lactation.

Bottlenose dolphins had a mean age of 12 in California and 17 in Florida (Figures S1A and S2A, and Table S1), while killer whales had a mean age of 11 in California, 17 in Florida, and 16 in Texas (Figures S1B and S2B, and Table S2). No significant differences in age were found by location in any of the species (Bottlenose dolphins—*t*-test: *p* > 0.05; killer whales—one-way ANOVA: *p* > 0.05).

### 3.2. Occurrence of PAEs in Delphinids

All six PAEs were detected in at least one individual bottlenose dolphin or killer whale (Tables S7 and S8). Killer whales displayed significantly higher mean ΣPAEs (5103 ng·mL^−1^) compared to bottlenose dolphins (562 ng·mL^−1^; *t*-test: t(72) = 2.87, *p* < 0.01; Tables S7 and S8), which is likely associated with a different variety of prey consumption and species-specific differences in metabolic transformation [47,48,66]. The likelihood of biomagnification was inconsequential as it has been demonstrated that PAEs do not biomagnify in aquatic food webs [66,67].

**Figure 1:**
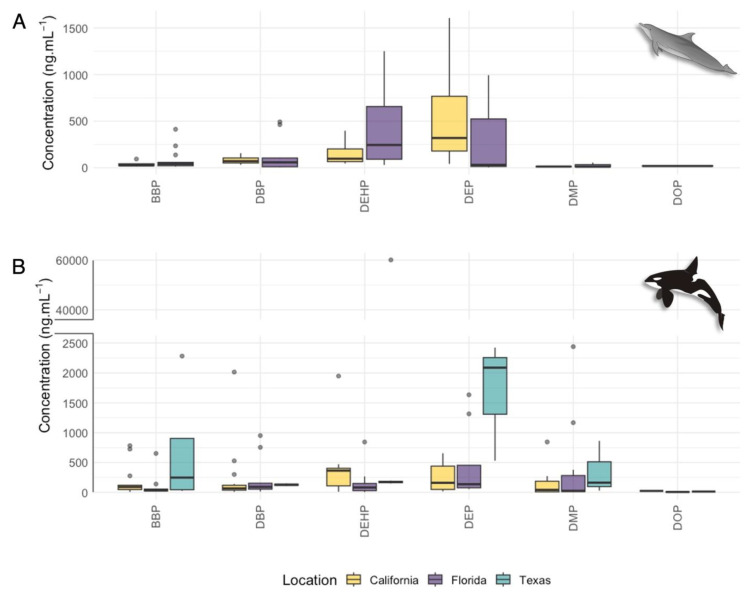
Phthalate esters (PAEs) concentrations (ng·mL^−1^) in serum samples of (**A**) bottlenose dolphins and (**B**) killer whales collected in three SeaWorld facilities (California, Florida, and Texas). Bottlenose dolphins were only sampled in two facilities (California and Florida). In the boxplots, the limit of the box closest to zero indicates the 25th percentile, the black line within the box indicates the median, and the limit of the box farthest from zero indicates the 75th percentile. The whiskers represent extreme observations, and the gray dots represent the outliers. A break in the *y*-axis is indicated by the gray bar. Individual dolphins may be represented multiple times in these plots as they were re-sampled over time.

The compounds with higher mean concentrations in bottlenose dolphins and killer whales were DEP (381 ng·mL^−1^ and 1483 ng·mL^−1^, respectively) and DEHP (305 ng·mL^−1^ and 2892 ng·mL^−1^, respectively; Figure 1A,B; 2892 ng·mL^−1^). DEP is a phthalate often added to cosmetics and personal care products [68], while DEHP is commonly used in commercially produced plastic items [69], and they are two of the most commonly used phthalates in commercial manufacturing. In fact, DEHP represented 37.1% of the global plasticizers market in 2015 [23]. Our findings align with previous studies where DEP and DEHP are the most common compounds found in cetacean species, independent of the matrix or area of study [30,31,70]. Moreover, studies assessing PAE metabolite concentrations have also detected DEP and DEHP metabolites in different cetacean species [31,32,33,34,36,37,71,72].

The compounds with lower concentrations were DMP (19.30 ng·mL^−1^) and DOP (19.30 ng·mL^−1^) in bottlenose dolphins (Table S7) and DOP (12.50 ng·mL^−1^) in killer whales (Table S8). DMP is used in manufacturing solid rocket propellant and consumer products such as insect repellents and plastics [73], and DOP is commonly used in synthetic rubbers [74]. The low concentrations of DMP and DOP that we observed are likely due to these compounds being used in lower proportions in plastic and personal care products and/or due to the absence of sources of these compounds in the localities of water and food supply.

Bottlenose dolphins displayed a higher PAE frequency variability by location (CA: 24% and FL: 43%; *t*-test: *p* > 0.05) than killer whales (CA: 61%, FL: 60%, and TX: 63%; one-way ANOVA: *p* > 0.05). Significant differences in BBP were found in bottlenose dolphins by location (*t*-test: t(31.83) = −2.99, *p* <0.05; Figure 1A). The relatively higher BBP concentrations in Florida bottlenose dolphins (mean of 85.60 ng·mL^−1^) compared to California (mean of 40.10 ng·mL^−1^) could likely be related to BBP sources where the food supply was gathered or from food packing or plastic containers where the food was stored.

DMP significantly decreased with age in bottlenose dolphins (Linear regression: F_1,34_ = 4.531, *p* < 0.05; Figure S3), which may be due to the “dilution effect”, where the larger the individual is, the lower the contaminant concentrations are [75]. Previous studies have shown that high molecular weight PAEs (i.e., BBP, DEHP, and DOP) and some low molecular weight PAEs (i.e., DBP and DEP) undergo trophic dilution in the marine food web [67,76,77]. However, to the best of our knowledge, it has not been clarified if the same occurs with DMP. We theorize that this may be true for bottlenose dolphins, as suggested by our results.

**Figure 2:**
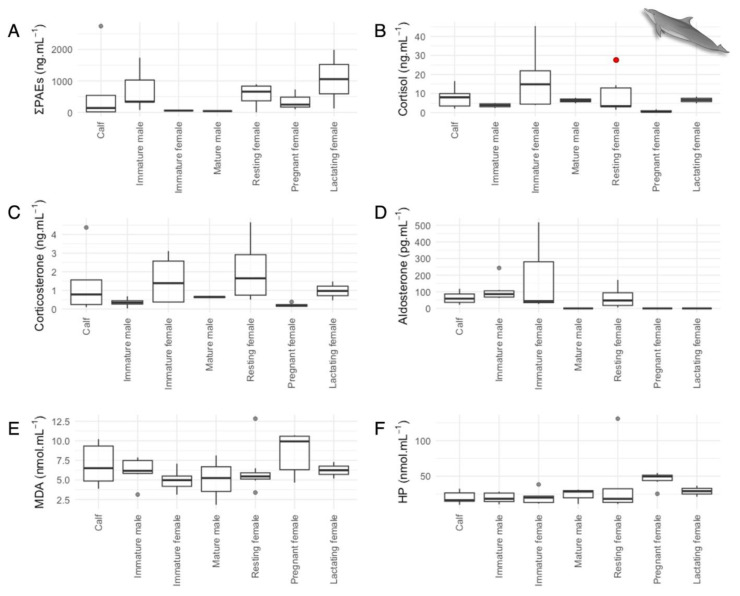
Variations in (**A**) sum of phthalate esters (PAEs), (**B**) cortisol, (**C**) corticosterone, (**D**) aldosterone, (**E**) MDA, and (**F**) hydrogen peroxide (HP) concentrations of bottlenose dolphin (*Tursiops truncatus*) serum samples by the different demographic statuses. Mature females were classified into three different categories: pregnant, lactating, and resting (when not pregnant or lactating). In the boxplots, the limit of the box closest to zero indicates the 25th percentile, the black line within the box indicates the median, and the limit of the box farthest from zero indicates the 75th percentile. The whiskers represent extreme observations, and the gray dots represent the outliers. Individual dolphins may be represented multiple times in these plots as they were re-sampled over time. The red dot indicates a resting female undergoing a known stressful event (raised on a crane for a medical procedure; total time out of the water was <1 h).

**Figure 3:**
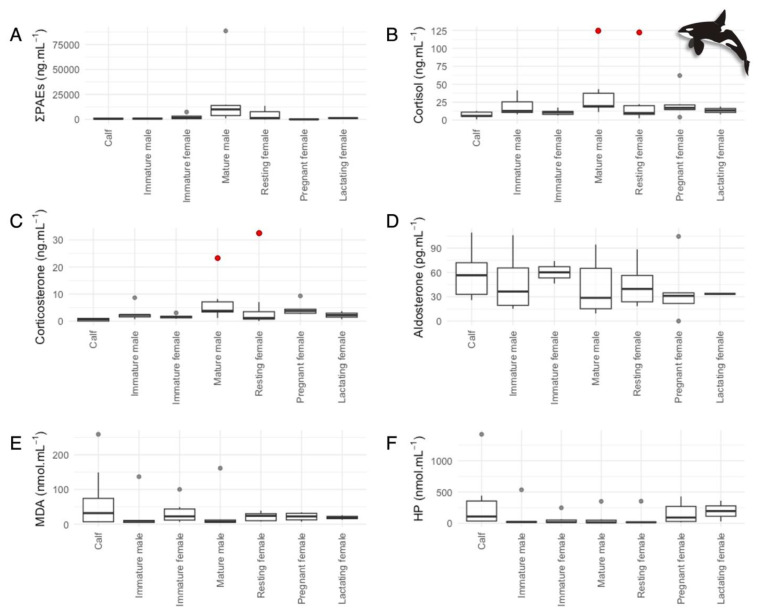
Variations in (**A**) sum of phthalate esters (PAEs), (**B**) cortisol, (**C**) corticosterone, (**D**) aldosterone, (**E**) MDA, and (**F**) hydrogen peroxide (HP) concentrations of killer whale (*Orcinus orca*) serum samples by the different demographic statuses. Mature females were classified into three different categories: pregnant, lactating, and resting (when not pregnant or lactating). In the boxplots, the limit of the box closest to zero indicates the 25th percentile, the black line within the box indicates the median, and the limit of the box farthest from zero indicates the 75th percentile. The whiskers represent extreme observations, and the gray dots represent the outliers. Individual animals may be represented multiple times in these plots as they were re-sampled over time. Red dots indicate a mature male and a resting female undergoing known stressful events (raised on a lifting floor for a health exam for 20 min).

The sum of PAEs significantly varied with maturity and reproductive stages in both species (bottlenose dolphins—one-way ANOVA: F_(1,6)_ = 2.76, *p* < 0.05; Figure 2A; killer whales—one-way ANOVA F_(1,6)_ = 4.45, *p* < 0.01; Figure 3A; Table S9). The Tukey Posthoc Test determined only significant differences between killer whale mature males and immature males (*p* < 0.001) and killer whale mature males and pregnant females (*p* < 0.001). These differences could be reflecting normal individual fluctuations in PAE concentrations or differences in physiological processes like metabolic rates and excretion/detoxification capabilities [32,37,78,79]. Pregnant females would also be experiencing volume expansion or dilution [75].

Compared with other studies on cetaceans worldwide, this study found consistently higher PAE concentrations for BBP, DEHP, DEP, and DMP (Table 1). However, it is important to acknowledge the limitations in making direct comparisons due to varying study matrices. Studies assessing cetacean PAEs are still scarce and no other studies on serum that would allow us to make a reliable comparison were identified. In a study pairing serum and urine concentrations of DEP and MEP (DEP metabolite) in DEP-exposed rats, urinary concentrations were 10^5^ times higher than in serum [80]. Thus, if the same occurs in cetaceans, we would expect even higher MEP or other metabolite concentrations in their urine, which would exceed concentrations found in previous studies. For instance, Dziobak et al. (2021) [32] noted a urinary MEP geometric mean concentration of 4.51 ng·mL^−1^ in bottlenose dolphins, while Hart et al. (2018) [37] reported 5.40 ng·mL^−1^ for MEP (against a mean DEP concentration of 381 ng·mL^−1^ in the present study) and 1.90 ng·mL^−1^ for MEHP (DEHP metabolite) in bottlenose dolphins (against a mean DEHP concentration of 305 ng·mL^−1^ in the present study; Table 1). Further research is needed to confirm if this ratio is similar in cetaceans.

### 3.3. PAEs Transference from Mother to Calf

**Figure 4:**
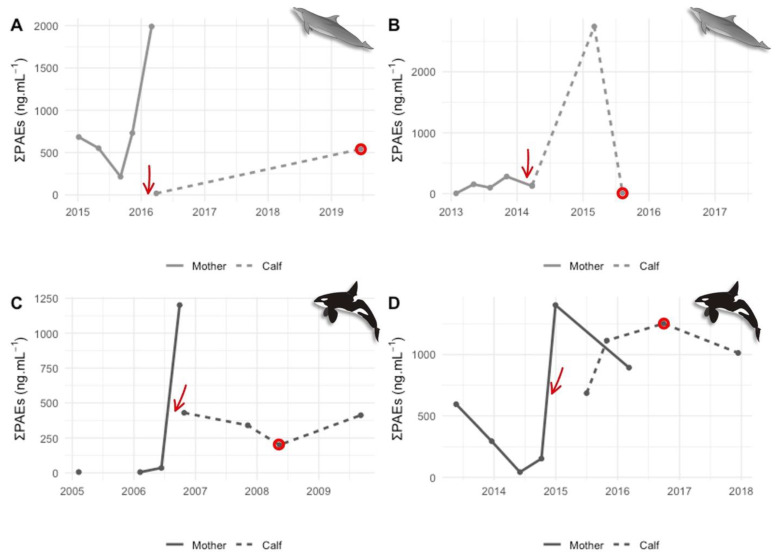
Temporal variation in the sum of phthalates (PAEs) in mother–calf pairs of bottlenose dolphins (**A**,**B**) and killer whales (**C**,**D**). Red arrows indicate parturition dates (**A**: 23 February 2016; **B**: 12 February 2014; **C**: 18 September 2006; and **D**: 2 December 2014). Red circles indicate sampling in calves after post-weaning events.

A time series analysis was conducted in two bottlenose dolphin (Figure 4A,B) and two killer whale mother–calf pairs (Figure 4C,D) to investigate PAE transference via the placenta and lactation.

All calves had detectable PAE concentrations, indicating potential transference through these pathways. Our findings align with human research, where phthalate metabolites have been detected in multiple maternal–placental–fetal compartments including maternal urine [82], amniotic fluid [83], placental tissue [84], umbilical cord blood [85], and meconium [86]. PAEs have also been found in breast milk [87], signifying transference via lactation in mammals. In fact, three of the calves in the present study exhibited gradual (A) or sharp (B and D) increases in ΣPAEs after birth and a subsequent progression and increase during lactation. Moreover, one of the dams (D) exhibited a decrease in PAE load with the birth of the calf and lactation. Unfortunately, the other females were not sampled after birth of the calves, thus preventing any further conclusions regarding this phenomenon. Nevertheless, our results suggest that lactation could represent a relevant route of PAE exposure for delphinid calves, contrary to previous discussions and theories [32,37,38].

In our study, only one of the calves (B) displayed higher concentrations than the mother, in contrast to previous research on other types of contaminants [88,89,90,91]. These results imply that different bioaccumulation and excretion mechanisms among these contaminant groups may occur. In fact, it has been described that phthalates do not accumulate in tissues and organs like other contaminants. In humans, they are quickly metabolized and excreted in urine and feces [92], and similar mechanisms are likely to be true in other mammals such as cetaceans. Even though phthalates do not tend to accumulate within the organism, they are continuously released into the marine environment, chronically exposing these individuals. Previous research has shown that dolphins have higher PAE metabolite average concentrations than human reference populations [32,36,38], suggesting that they either have higher excretion rate capabilities or higher PAE concentrations to be excreted.

Studies on mother–calf contaminant transference in cetaceans are still scarce, especially in emerging contaminants such as PAEs. To the best of our knowledge, only one previous research has assessed phthalate concentrations in mother–calf pairs of bottlenose dolphins [37], detecting urinary phthalate metabolites in both groups but unable to determine if calves were nursing during sampling. This knowledge gap contrasts with evidence of PAE transference through placenta and lactation in humans [82,83,84,85,86]. Our study provides insight into potential PAE transference from mothers to calves in bottlenose dolphins and killer whales.

### 3.4. Hormone Profiles in Delphinids

In this study, two bottlenose dolphins and two killer whales underwent a known stressful event (IUKSE) by being raised on a lifting floor or out of the water for a health exam. Animals had their blood drawn before and after the events, which lasted < 1 h for bottlenose dolphins and 30 min for killer whales. An increase in all hormones was observed in both species after the stressful events (Figure S4).

Killer whales displayed 2.7 to 3.5 times higher mean concentrations of cortisol and corticosterone (cortisol range: 0.90–124 ng·mL^−1^; corticosterone: 0.03–32.7 ng·mL^−1^) compared to bottlenose dolphins (cortisol range: 0.22–45.4 ng·mL^−1^; corticosterone: 0.03–4.65 ng·mL^−1^), respectively (*t*-test: *p* < 0.05; Figure 3B,C and Figure 4B,C; Table S10). In contrast, bottlenose dolphins exhibited a 1.4 times higher aldosterone mean concentration (range: 0.01–518 ng·mL^−1^) than killer whales (range: 0.01–109 ng·mL^−1^; *t*-test: *p* > 0.05; Figure 3D and Figure 4D; Table S10). Because IUKSEs could represent a confounding factor, these individuals were excluded from the following statistical analyses. Some hormone profiles still varied significantly by species when excluding IUKSEs (cortisol *t*-test: t(64) = 4.83, *p* < 0.001; corticosterone *t*-test: t(67) = 4.24, *p* < 0.001; aldosterone *t*-test: *p* > 0.05), which is likely related to their unique species-specific features like body size, ecological niche, diet, and adrenal hormone regulation. Another potential explanation for these results is that the processing method as an extraction step prior to assay was required for killer whale samples only. This process likely liberated more bound hormones and was a measure of total hormones (free + bound) but may only have quantified free hormones in bottlenose dolphins.

Corticosterone concentrations were notably lower than cortisol concentrations, suggesting a predominance of cortisol in both species. The ratio of mean cortisol–corticosterone found in bottlenose dolphins was 7:1 and in killer whales was 5:1, which aligns well with previous studies on bottlenose dolphins (ratio of 5:1 [93,94]) and killer whales (ratio of 4.7:1 [95]).

Aldosterone concentrations varied by location in bottlenose dolphins only (*t*-test: t(25) = 3.62, *p* < 0.01; Figure S2A), with a higher mean concentration in California (122 ng·mL^−1^) than in Florida (25.1 ng·mL^−1^), which could be related to dietary differences or differences in salinity [40,43,96,97,98,99]. In fact, the average salinity in the California site (33.59) for all the sampled years was higher than in the Florida (27.60) or Texas (27.69) locations. However, it is still unknown what the annual, circadian, and life history concentration ranges for aldosterone in bottlenose dolphins are, and the observed concentrations might be within the population range. Thus, further investigation should be conducted to determine the aldosterone base levels in the species. Another important factor to consider is that due to the low sample size, aldosterone was only quantified in 27 out of the 41 bottlenose dolphin samples and 30 out of the 44 killer whale samples.

Significant differences in cortisol concentrations were found among the different demographic statuses of bottlenose dolphins (one-way ANOVA: F_(6,27)_ = 7.08, *p* < 0.001), with pregnant females differing from calves, immature females, resting females, and mature males (Tukey Test: *p* < 0.05; Figure 2B; Table S9). Interestingly, pregnant females displayed the lowest concentrations of all demographic statuses (range: 0.22–1.76 ng·mL^−1^). This contrasts with previous work in bottlenose dolphins that have shown cortisol increases during late gestation, in particular in the last month [47]. It is well known that cortisol plays a crucial role in the later stages of gestation for the development and maturation of the respiratory system in mammals [100,101], thus the absence of sampling immediately before birth (our late gestation sampling occurred at 9 months post-conception) likely prevented us from observing this increase.

Significant differences in cortisol (one-way ANOVA: F_(6,33)_ = 2.56, *p* < 0.05) and corticosterone (one-way ANOVA: F_(6,33)_ = 4.72, *p* < 0.01) concentrations were also found among killer whale demographic statuses. Mature males displayed significantly higher cortisol and corticosterone concentrations than calves (Tukey Test: cortisol—*p* < 0.05; corticosterone—*p* < 0.01; Figure 4B,C; Table S9), and pregnant females also exhibited significantly higher corticosterone concentrations than calves (Tukey Test: *p* < 0.01; Figure 3C; Table S9). Our results are aligned with a previous study on killer whales [52]. GC concentrations are typically elevated in sexually mature individuals as they are involved in competitive and sexual interactions [102,103,104] and in pregnant females, as they undergo unique physiological changes and metabolic and energetic demands [105].

Aldosterone concentrations also varied significantly within the bottlenose dolphin demographic statuses (one-way ANOVA: F_(6,18)_ = 32.33, *p* < 0.001), with mature males and lactating and pregnant females displaying lower concentrations than calves, immature individuals, and resting females (Tukey Test: *p* < 0.05). Fair et al. (2014) have reported similar findings in wild bottlenose dolphins from South Carolina and Florida, US, with juvenile dolphins exhibiting significantly higher aldosterone concentrations compared to adults [106]. It has also been well-documented in humans that plasma aldosterone concentrations are highest in newborns and lowest in the elderly population [107].

Considering the hormonal temporal variations in the four mother–calf pairs of both species (Figure S5), we could surmise that there is not a clear pattern in cortisol, corticosterone, or aldosterone concentrations throughout the pregnancies. An increase in cortisol concentrations was only observed in a killer whale (pair 4), which occurred ~3 months before the parturition date. Sampling closer to the parturition date would likely show an increase in the hormone concentration. These results are aligned with previous research on bottlenose dolphins [47,95] and killer whales [53].

A sharp increase in cortisol and corticosterone concentrations was observed in bottlenose dolphins (Figure S5A,B,E,F) throughout the lactation period, which is considered the most energetically demanding phase of the female reproductive cycle [105]. Cortisol concentrations also increased in calves, in three out of the four pairs (Figure S5A,B,D), which may be related to weaning events [103].

Notably, there is an overall similarity between the steroid hormone patterns (Figure S5). In fact, we found positive linear correlations between cortisol and corticosterone in bottlenose dolphins (F_1,27_ = 44.46, *p* < 0.001—Figure S6A) and killer whales (F_1,38_ = 92.13, *p* < 0.001—Figure S6D) and between cortisol and aldosterone in bottlenose dolphins only (F_1,23_ = 10.67, *p* < 0.01—Figure S6B). Cortisol and corticosterone are both GC hormones, produced in the adrenal glands and activated in response to the adrenocorticotropic hormone (ACTH; [40]). This correlation is well known and has been previously observed in cetaceans [95,108]. Cortisol and aldosterone, in turn, can also be related as there can be a concomitant increase in circulating aldosterone concentrations during a stress response [40,43,109,110]. Cortisol influences the renin–angiotensin–aldosterone system (RAAS) by stimulating renin release, angiotensinogen production, and angiotensin-converting enzyme (ACE) activity, all of which will promote aldosterone release [111,112]. Previous research on cetaceans has also described correlations between GCs and aldosterone killer whales [95] and bottlenose dolphins [113]. The limited number of samples in this study might have masked any potential correlations between GCs and aldosterone in killer whales.

### 3.5. Oxidative Stress in Delphinids

Mean MDA and HP concentrations were significantly higher (MDA *t*-test: t(50) = 6.42, *p* < 0.001; HP *t*-test: t(54) = 3.18, *p* <0.01) in killer whales (MDA: 35.3 nmol·mL^−1^—Figure 3E; HP: 139 nmol·mL^−1^—Figure 3F) than in bottlenose dolphins (MDA: 6.43 nmol·mL^−1^—Figure 2E; HP: 28.1 nmol·mL^−1—^Figure 2F; Tables S9 and S11). These results could be associated with the differentiated diets and physiology of the species or with the higher PAE exposure found in killer whales.

Both oxidative stress biomarkers varied significantly with location in bottlenose dolphins (MDA *t*-test: t(31) = −2.56 *p* < 0.05; HP *t*-test: t(29) = −2.47, *p* <0.05; Figure S2A) and in killer whales (MDA one-way ANOVA: F_(2,37)_ = 3.87, *p* < 0.05; HP one-way ANOVA: F_(2,37)_ = 5.36, *p* < 0.01; Figure S2B). Higher concentrations of MDA and HP were found in Florida delphinids (Table S11; Figure S2A,B), indicating that the individuals in this location may be facing increased free radicals and cellular damage. ROS and cellular damage levels increase with age [114,115]; however, we did not find any correlations between HP or MDA and age in any of the species. Other factors known to cause cellular damage in mammals that could be affecting these Florida dolphins include genetic factors [116], nutritional deficiencies [117,118], inflammation [119], infection [120,121], and chemical exposure to a wide variety of contaminants [122,123]. Further investigation is warranted to better understand the specific factors contributing to the relatively higher oxidative stress in these individuals. No significant correlations were found between the oxidative stress biomarkers and the demographic statuses of any of the species (one-way ANOVA: *p* > 0.05).

Positive linear correlations were found between HP and MDA in both species (bottlenose dolphins: F1,32 = 31.69, *p* < 0.001—Figure S7A; killer whales: F1,38 = 41.90, *p* < 0.001—Figure S7B), suggesting that increased levels of HP may be contributing to the observed cellular damage, which is supported by previous research in human cells [124].

### 3.6. Linear Mixed Models

Linear mixed model analyses were carried out for each of the five stress biomarkers, and model selection was based on the lowest AIC. Three of the models were statistically significant. The cortisol model 1 in bottlenose dolphins (AIC = 85.11, df = 36, R^2^m = 0.43, R^2^c = 1.00, Table S12), aldosterone model 4 (AIC = 62.65, df = 31, R^2^m = 0.99, R^2^c = 0.99), and HP model 10 (AIC = 90.53, df = 38, R^2^m = 0.42, R^2^c = 1.00) in killer whales. Significant parameter estimates included month, year, HP, and MDA in the cortisol model 1 in bottlenose dolphins; month, location, age, and ΣPAEs in the aldosterone model 4 in killer whales; and month, year, and status in the HP model 10 in killer whales (Table 2).

The cortisol model 1 included the variables month, year, location, age, status, ΣPAEs, HP, and MDA as fixed effects and dolphin ID as random effects (Table 2). Month, year, HP, and MDA were significant and explained 43% of the cortisol variability observed in bottlenose dolphins (Table S12). This number increases to 100% when also considering dolphin ID.

The aldosterone model 4 included the variables month, year, location, age, status, and ΣPAEs (Table 2). The variables that were significant in this model were month, year, location, status, and ΣPAEs, which explained 99% of the aldosterone variability observed in killer whales (Table S12). The percentage is the same when including dolphin ID.

The HP model 10 included month, year, location, age, and status, with month, year, and status being significant. These variables explained 42% of the HP variability observed in killer whales and increased to 100% when including dolphin ID (Table S12).

It was interesting to note that even though ΣPAEs had no direct association with aldosterone, it caused a significant effect on the aldosterone concentrations of killer whales according to the model results (model 4; Table 2), suggesting an indirect influence or an intricate mechanism at play. Previous studies have described phthalates as endocrine disruptors that can cause a decrease in aldosterone production [20,125,126], which could explain its significance in our findings.

## 4. Conclusions and Future Studies

This study highlights the significance of a multidisciplinary approach for a better understanding of potential correlations between physiological stress and plasticizers on cetaceans. This study marks the first investigation of potential correlations between PAEs, hormones, and oxidative stress biomarkers and provides evidence that PAEs may induce physiological responses in cetaceans.

Our results indicated PAEs exposure in both killer whales and bottlenose dolphins across three aquariums, with killer whales displaying higher concentrations for all PAEs, except DOP. The most prevalent PAEs in both species were DEP and DEHP, which are two of the most commonly used phthalates in commercial manufacturing and the most predominantly found in other cetaceans. PAEs were also detected in newborn bottlenose dolphins and killer whales, indicating potential transference through placenta and nursing.

Hormone concentrations varied by species and demographic status. Aldosterone concentrations also varied by location in bottlenose dolphins, with higher concentrations found in California. Cortisol and corticosterone were positively correlated in both species, while cortisol and aldosterone were only correlated in bottlenose dolphins, suggesting a potential association with stress responses. Individuals from Florida also exhibited elevated oxidative stress biomarker concentrations, reflecting the need for further investigations into factors influencing oxidative stress. HP and MDA were also positively correlated in both species, indicating that increased HP levels are likely contributing to cellular damage, potentially impairing their defense mechanisms.

Significant correlations between aldosterone, month, year, location, demographic status, ΣPAEs, and killer whale ID were found, with aldosterone concentration variability being explained by these variables at a rate of 99%. This is an impressive level of explanatory power and suggests that PAEs could be acting as endocrine disruptors in these individuals and influencing their aldosterone concentrations. Nonetheless, it is important to highlight that such a high level of explanatory power may indicate data overfitting and may not adequately account for other factors not assessed in our study.

It is also important to stress that we only evaluated a limited number of compounds and animals in this study. Our previous study on 30 per- and polyfluoroalkyl substances (PFAS) in these species found that PFAS were significantly correlated with ROS production in killer whales. Therefore, further investigation including other contaminant classes, compounds, and larger sample size is necessary for a better understanding of their cumulative and synergistic effects on these individuals. Moreover, controlled experiments, mechanistic studies, and comprehensive exposure assessments are necessary to validate these findings and elucidate underlying causal mechanisms.

This research lays the groundwork for future studies, providing a holistic perspective on the potential impacts of PAEs on the health and physiology of these delphinids. Such assessments may be used to aid in the well-being of aquarium-based delphinids and in the conservation and management efforts of wild individuals.

## Figures and Tables

**Table 1:** Mean ± standard deviation (and range) of phthalate concentrations in varied matrices of different cetacean species worldwide. ND: non-detected. LOQ: limit of quantification.

Study	Species	Matrix (Unit)	n	BBP	DBP	DEHP	DEP	DMP	DOP
This study	*Tursiops truncatus*	Serum (ng·mL^−1^ ww)	36	74.90 ± 104.0 (12.80–413.0)	116.0 ± 153.0 (6.00–492.0)	305.0 ± 323.0 (30.30–1251)	381.0 ± 490.0 (5.540–1607)	19.30 ± 18.40 (7.680–56.20)	19.30 ± 6.910 (14.40–24.10)
	*Orcinus orca*			210.0 ± 439.0 (7.510–2281)	835.0 ± 2314 (8.160–12,130)	2892 ± 10,523 (5.370–60,097)	1483 ± 3165 (15.30–13,304)	506.0 ± 1296 (5.180–6433)	12.50 ± 8.220 (6.220–24.00)
Fossi et al. (2016) [71]	*Balaenoptera physalus*	Skin (ng·g^−1^ dw)	40	-	-	ND	-	-	-
Baini et al. (2017) [31]	*Balaenoptera physalus*	Skin (ng·g^−1^ dw)	3	-	-	7051	-	-	-
	*Tursiops truncatus*		1	-	-	26,068	-	-	-
	*Grampus griseus*		1	-	-	1130	-	-	-
	*Stenella coeruleoalba*		2	-	-	21,460	-	-	-
Montoto-Martinez et al. (2021) [30]	*Tursiops truncatus* *Stenella coeruleoalba* *Globicephala macrorhynchus* *Grampus griseus* *Kogia breviceps* *Lagenodelphis hosei*	Muscle (ng·g^−1^)	2 5 1 2 1 1	-	-	(102–1533)	(13–225)	-	-
Routti et al. (2021) [70]	*Balaenoptera musculus*	Blubber (ng·g^−1^ ww)	7	<10	-	20	<37.5	-	-
	*Balaenoptera physalus*		6	<10	-	42	<37.5	-	-
	*Balaena mysticetus*		5	<10	-	ND	<37.5	-	-
Andvik et al. (2024) [38]	*Physeter macrocephalus*	Blubber (ng·g^−1^ ww)	1	<0.2	-	4.1	<3.0	-	<0.3
	*Globicephala melas*		1	1.7	-	<45	<14	-	<3.0
	*Lagenorhynchus albirostris*		1	<0.2	-	120	<3.0	-	205
	*Phocoena phocoena*		1	<0.2	-	23	<3.0	-	65
	*Orcinus orca (stranded)*		2	<0.2–<0.6	-	(ND–3.3)	<14–<0.5	-	ND–<0.3
	*Orcinus orca* (free-living)		9	<0.6–5.3	-	(<45–55)	<15	-	<3.0
Sambolino et al. (2024) [81]	*Globicephala macrorhynchus*	Blubber (ng·g^−1^ ww)	15	ND–10.41	ND–284.1	ND–93.63	<LOQ–93.24	ND–25.98	-
	*Tursiops truncatus*		9	<LOQ–57.11	ND–717.6	127.3–517.8	ND–38.94	ND	-

**Table 2:** ANOVA results from the statistically significant selected linear mixed models. Asterisks indicate significance at the 0.05 (*), 0.01 (**), and 0.001 (***) levels.

Species	Biomarker and Model	Variable	F Statistics_df_	*p* Value
*T. truncatus*	Cortisol Model 1	Month	F_1,11_ = 10,048	*p* < 0.001 ***
		Year	F_1,12_ = 15,459	*p* < 0.001 ***
		Location	F_1,1_ = 16.310	*p* = 0.056
		Age	F_1,1_ = 3.7177	*p* = 0.194
		Status	F_1,5_ = 15.324	*p* = 0.062
		ΣPAEs	F_1,1_ = 0.0392	*p* = 0.861
		HP	F_1,1_ = 50.091	*p* = 0.019 *
		MDA	F_1,1_ = 23.111	*p* = 0.041 *
*O. orca*	Aldosterone Model 4	Month	F_1,10_ = 298.24	*p* = 0.003 **
		Year	F_1,11_ = 113.31	*p* = 0.009 **
		Location	F_1,2_ = 213.83	*p* = 0.005 **
		Age	F_1,1_ = 518.59	*p* = 0.999
		Status	F_1,3_ = 190.49	*p* = 0.005 **
		ΣPAEs	F_1,1_ = 351.76	*p* = 0.034 *
*O. orca*	Hydrogen peroxide Model 10	Month	F_1,11_ = 240.50	*p* = 0.004 **
		Year	F_1,15_ = 300.50	*p* = 0.003 **
		Location	F_1,2_ = 4.0899	*p* = 0.107
		Age	F_1,1_ = 0.0011	*p* = 0.975
		Status	F_1,6_ = 31.0292	*p* = 0.025 *

## Data Availability

The data will become available upon request.

## Supplementary Materials

The following supporting information can be downloaded at: https://www.mdpi.com/article/10.3390/ani14101488/s1, Table S1: Demographic status assignment of each sample of the bottlenose dolphins (*Tursiops truncatus*) analyzed in this study; Table S2: Demographic status assignment of each sample of the killer whales (*Orcinus orca*) analyzed in this study; Table S3: Gas chromatograph (GC) column/oven conditions; Table S4: Mass spectrometer (MS) parameters for gas chromatography (GC)-MS analysis; Table S5: Summary of the method for the phthalates analysis, including compounds, retention time (RT; in minutes), and ions monitored (m/z); Table S6: Results for the phthalates (PAEs) method validation, including linearity (R^2^), the method detection limit (MDL; in ng·mL^−1^), intra- and inter-day relative standard deviation (RSD), average analyte recovery (%), and matrix effect; Table S7: Mean ± standard deviation, range, and detection frequency (F; %) of phthalate ester (PAEs) concentrations (ng·mL^−1^) in bottlenose dolphins by sampling location; Table S8: Mean ± standard deviation, range, and detection frequency (F; %) of phthalate ester (PAEs) concentrations (ng·mL^−1^) in killer whales by sampling location; Table S9: Mean concentration ± standard deviation, range of total phthalates (ΣPAEs; ng·mL^−1^), cortisol (ng·mL^−1^), corticosterone (ng·mL^-1^), aldosterone (pg·mL^−1^), MDA (malondialdehyde-metric for TBARS; nmol·mL^−1^) and hydrogen peroxide (HP; nmol·mL^−1^) concentrations, and sample size (N) by demographic state of bottlenose dolphins (*Tursiops truncatus*) and killer whales (*Orcinus orca*); Table S10: Mean ± standard deviation, range of the concentrations of the hormones cortisol (ng·mL^−1^), corticosterone (ng·mL^−1^), and aldosterone (pg·mL^−1^), and sample size (N) of delphinids by sampling location; Table S11: Mean ± standard deviation, range of the concentrations of the oxidative stress biomarkers MDA (malondialdehyde – metric for TBARS; nmol·mL^−1^) and hydrogen peroxide (HP; nmol·mL^−1^), and sample size (N) of delphinids by sampling location; Table S12: Linear mixed model (LMM) selection parameters of bottlenose dolphin (*Tursiops truncatus*) and killer whale (*Orcinus orca*) blood stress-related hormone (cortisol, corticosterone, and aldosterone), and oxidative stress biomarker (hydrogen peroxide – HP, and malondialdehyde - MDA) concentrations relative to month, year, location, age, demographic status, and sum of phthalates (ΣPAEs); Table S13: Parameter estimates from the statistically significant selected linear mixed models for bottlenose dolphin (*Tursiops truncatus*; cortisol model 1) and killer whale (*Orcinus orca*; aldosterone model 4 and hydrogen peroxide model 10) serum concentrations; Figure S1: Distribution of the numeric variables analyzed from serum samples of bottlenose dolphins (A) and killer whales (B) from three SeaWorld facilities (California, Florida, and Texas), including age, hormones, oxidative stress biomarkers, 6 compounds of PAEs and the sum of PAEs (ΣPAEs); Figure S2: Boxplots of age, hormones (cortisol, corticosterone, and aldosterone), and oxidative stress biomarkers (HP and MDA) in bottlenose dolphins (A) and killer whales (B) by SeaWorld facilities (California, Florida, and Texas). Bottlenose dolphins were only sampled in two facilities (California and Florida); Figure S3: Significant linear correlation between age (log + 1) and DMP (log + 1) in bottlenose dolphins; Figure S4: Temporal variation of the hormones cortisol (A and D), corticosterone (B and E), and aldosterone (C and F) in mature individuals of bottlenose dolphins (n = 2) and killer whales (n = 2) before and after being raised on a lifting floor or out of the water for a medical procedure (i.e., stressful event); Figure S5: Temporal variation of steroid hormones (cortisol, corticosterone, and aldosterone) in mother-calf pairs of bottlenose dolphins (A, B, E, F, I, and J) and killer whales (C, D, G, H, K, and L); Figure S6: Linear correlations between steroid hormones (cortisol, corticosterone, and aldosterone; log+1) in bottlenose dolphins (A, B, and C) and killer whales (D, E, and F); Figure S7: Linear correlations between the oxidative stress biomarkers hydrogen peroxide (HP) and malondialdehyde (MDA; log+1) in bottlenose dolphins (A) and killer whales (B).
