# Supplementary material for: Assessment of Phthalate Esters and Physiological Biomarkers in Bottlenose Dolphins (Tursiops truncatus) and Killer Whales (Orcinus orca)
Source: Animals (Basel). 2024 May 17;14(10):1488. doi: 10.3390/ani14101488 (PMC11117373; doi:10.3390/ani14101488)
Supplement: Supplementary file 1 [file animals-14-01488-s001.zip › animals-2963161-supplementary.docx]

**Assessment of phthalate esters and physiological biomarkers in**

**bottlenose dolphins (*Tursiops truncatus*) and killer whales (*Orcinus* orca)**

Leila S. Lemos^1,2*^, Amanda DiPerna^2^, Karen J. Steinman^3^, Todd R. Robeck^3,4^, Natalia Quinete^1,2*^

^1^ Institute of Environment, Florida International University, North Miami, FL, 33181, USA

^2^Emerging Contaminants of Concern Research Laboratory, Department of Chemistry & Biochemistry, College of Arts, Sciences, and Education, Florida International University, North Miami, FL, 33181, USA

^3^ SeaWorld & Busch Gardens Species Preservation Laboratory, United Parks and Resorts, San Diego, CA, 92109, USA

^4^ United Parks and Resorts, 7007 Sea Harbor Drive, Orlando Florida 32821

**Corresponding authors:**

Leila S. Lemos: [leslemos@hotmail.com](mailto:leslemos@hotmail.com)

Natalia Quinete: [nsoaresq@fiu.edu](mailto:nsoaresq@fiu.edu)

**Supplementary Information**

Table S1: Demographic status assignment of each sample of the bottlenose dolphins (*Tursiops truncatus*) analyzed in this study. Mature females are classified into three categories: pregnant, lactating, and resting (when not pregnant or lactating).

| **Sample #** | **Code** | **Location** | **Sampling Date** | **Demographic Status** | **Age** |
| --- | --- | --- | --- | --- | --- |
| 1 | Ttg 8326/56517 | Orlando, FL | 6-Jan-15 | Resting female (MC pair 1) | 31.84 |
| 2 | Ttg 8326/56517 | Orlando, FL | 30-Apr-15 | Pregnant female (MC pair 1) | 32.15 |
| 3 | Ttg 8326/56517 | Orlando, FL | 4-Sep-15 | Pregnant female (MC pair 1) | 32.50 |
| 4 | Ttg 8326/56517 | Orlando, FL | 11-Nov-15 | Pregnant female (MC pair 1) | 32.68 |
| 5 | Ttg 8326/56517 | Orlando, FL | 1-Mar-16 | Lactating female (MC pair 1) | 32.99 |
| 6 | Ttg 8326/56517 | Orlando, FL | 13-Dec-17 | Resting female (MC pair 1) | 34.77 |
| 7 | Tt1601/91595 | Orlando, FL | 28-Mar-16 | Calf (MC pair 1) | 0.09 |
| 8 | Tt1601/91595 | Orlando, FL | 19-Jun-19 | Calf (MC pair 1) | 3.32 |
| 9 | Tt9677/56520 | Orlando, FL | 28-Jan-13 | Resting female (MC pair 2) | 16.76 |
| 10 | Tt9677/56520 | Orlando, FL | 5-May-13 | Pregnant female (MC pair 2) | 17.02 |
| 11 | Tt9677/56520 | Orlando, FL | 4-Aug-13 | Pregnant female (MC pair 2) | 17.27 |
| 12 | Tt9677/56520 | Orlando, FL | 4-Nov-13 | Pregnant female (MC pair 2) | 17.52 |
| 13 | Tt9677/56520 | Orlando, FL | 25-Mar-14 | Lactating female (MC pair 2) | 17.91 |
| 14 | Tt1401/66869 | Orlando, FL | 25-Mar-14 | Calf (MC pair 2) | 0.11 |
| 15 | Tt1401/66869 | Orlando, FL | 4-Mar-15 | Calf (MC pair 2) | 1.05 |
| 16 | Tt1401/66869 | Orlando, FL | 8-Aug-15 | Calf (MC pair 2) | 1.48 |
| 17 | Tt1401/66869 | Orlando, FL | 9-May-17 | Calf (MC pair 2) | 3.24 |
| 18 | Tt0178x/48363 | San Diego, CA | 5-Oct-13 | Resting female (SE 1) | 12.17 |
| 19 | Tt0178x/48363 | San Diego, CA | 12-Oct-13 | Resting female (SE 1) | 12.19 |
| 20 | Tt9851/56507 | Orlando, FL | 9-Jan-15 | Resting female (SE 2) | 16.56 |
| 21 | Tt9851/56507 | Orlando, FL | 3-Feb-15 | Resting female (SE 2) | 16.63 |
| 22 | Tt0030/48027 | San Diego, CA | 7-Feb-05 | Immature male | 4.32 |
| 23 | Tt0030/48027 | San Diego, CA | 22-Feb-08 | Immature male | 7.36 |
| 24 | Tt0030/48027 | San Diego, CA | 15-Feb-10 | Immature male | 9.34 |
| 25 | Tt0027/48024 | San Diego, CA | 17-Nov-04 | Immature male | 4.49 |
| 26 | Tt0027/48024 | San Diego, CA | 19-Dec-05 | Immature male | 5.58 |
| 27 | Tt0027/48024 | San Diego, CA | 12-Dec-12 | Immature male | 12.56 |
| 28 | Tt0927x/53232 | San Diego, CA | 6-Feb-13 | Immature female | 3.65 |
| 29 | Tt0927x/53232 | San Diego, CA | 12-Jan-16 | Immature female | 6.58 |
| 30 | Tt0827/49235 | San Diego, CA | 22-Mar-11 | Immature female | 2.31 |
| 31 | Tt0827/49235 | San Diego, CA | 30-Apr-14 | Immature female | 5.42 |
| 32 | Tt0827/49235 | San Diego, CA | 4-Mar-20 | Immature female | 11.26 |
| 33 | Tt8737/48013 | San Diego, CA | 12-Jun-18 | Mature male | 42.44 |
| 34 | Tt8976/48032 | San Diego, CA | 14-Jul-10 | Mature male | 20.93 |
| 35 | Tt8976/48032 | San Diego, CA | 4-Feb-10 | Mature male | 20.49 |
| 36 | Tt9118B/48019 | San Diego, CA | 11-Jun-19 | Resting female | 29.19 |

Legend: MC: mother-calf pair (n = 2); SE: individual underwent stressful event (before and after, n = 2).

Table S2: Demographic status assignment of each sample of the killer whales (*Orcinus orca*) analyzed in this study. Mature females are classified into three categories: pregnant, lactating, and resting (when not pregnant or lactating).

| **Sample #** | **Code** | **Location** | **Sampling Date** | **Demographic Status** | **Age** |
| --- | --- | --- | --- | --- | --- |
| 1 | Oo7806/56408 | Orlando, FL | 8-Feb-05 | Resting female (MC pair 1) | 28.69 |
| 2 | Oo7806/56408 | Orlando, FL | 6-Sep-05 | Pregnant female (MC pair 1) | 29.26 |
| 3 | Oo7806/56408 | Orlando, FL | 7-Feb-06 | Pregnant female (MC pair 1) | 29.69 |
| 4 | Oo7806/56408 | Orlando, FL | 13-Jun-06 | Pregnant female (MC pair 1) | 30.03 |
| 5 | Oo7806/56408 | Orlando, FL | 29-Sep-06 | Lactating female (MC pair 1) | 30.33 |
| 6 | Oo0601/57052 | Orlando, FL | 26-Oct-06 | Calf (MC pair 1) | 0.10 |
| 7 | Oo0601/57052 | Orlando, FL | 7-Nov-07 | Calf (MC pair 1) | 1.14 |
| 8 | Oo0601/57052 | Orlando, FL | 13-May-08 | Calf (MC pair 1) | 1.65 |
| 9 | Oo0601/57052 | Orlando, FL | 7-Sep-09 | Calf (MC pair 1) | 2.97 |
| 10 | Oo0426/48000 | San Diego, CA | 22-May-13 | Resting female (MC pair 2) | 8.42 |
| 11 | Oo0426/48000 | San Diego, CA | 18-Dec-13 | Pregnant female (MC pair 2) | 8.99 |
| 12 | Oo0426/48000 | San Diego, CA | 3-Jun-14 | Pregnant female (MC pair 2) | 9.45 |
| 13 | Oo0426/48000 | San Diego, CA | 8-Oct-14 | Pregnant female (MC pair 2) | 9.80 |
| 14 | Oo0426/48000 | San Diego, CA | 30-Dec-14 | Lactating female (MC pair 2) | 10.02 |
| 15 | Oo0426/48000 | San Diego, CA | 8-Mar-16 | Resting female (MC pair 2) | 11.21 |
| 16 | Oo1426/79377 | San Diego, CA | 2-Jul-15 | Calf (MC pair 2) | 0.58 |
| 17 | Oo1426/79377 | San Diego, CA | 29-Oct-15 | Calf (MC pair 2) | 0.91 |
| 18 | Oo1426/79377 | San Diego, CA | 28-Sep-16 | Calf (MC pair 2) | 1.82 |
| 19 | Oo1426/79377 | San Diego, CA | 13-Dec-17 | Calf (MC pair 2) | 3.03 |
| 20 | Oo8876/56411 | Orlando, FL | 31-Mar-15 | Resting female (SE 1) | 26.34 |
| 21 | Oo8876/56411 | Orlando, FL | 31-Mar-15 | Resting female (SE 1) | 26.34 |
| 22 | Oo9377/68405 | San Antonio, TX | 24-Mar-15 | Mature male (SE 2) | 23.25 |
| 23 | Oo9377/68405 | San Antonio, TX | 24-Mar-15 | Mature male (SE 2) | 23.25 |
| 24 | Oo0127/47999 | San Diego, CA | 16-Sep-05 | Immature male | 4.04 |
| 25 | Oo0127/47999 | San Diego, CA | 11-Sep-15 | Immature male | 14.03 |
| 26 | Oo9378/48005 | San Antonio, TX | 10-Feb-97 | Immature male | 4.02 |
| 27 | Oo9378/48005 | San Diego, CA | 18-Jan-00 | Immature male | 6.96 |
| 28 | Oo9378/48005 | San Diego, CA | 13-Feb-15 | Immature male | 22.03 |
| 29 | Oo9126/68404 | San Diego, CA | 29-Aug-94 | Immature female | 3.14 |
| 30 | Oo9126/68404 | San Diego, CA | 14-Jul-97 | Immature female | 6.02 |
| 31 | Oo9126/68404 | San Diego, CA | 11-Jul-00 | Immature female | 9.01 |
| 32 | Oo0701/56714 | Orlando, FL | 1-Mar-10 | Immature female | 2.97 |
| 33 | Oo0701/56714 | Orlando, FL | 10-Apr-13 | Immature female | 6.08 |
| 34 | Oo0701/56714 | Orlando, FL | 19-May-16 | Immature female | 9.19 |
| 35 | Oo9426/47998 | San Diego, CA | 14-Aug-01 | Mature male | 23.62 |
| 36 | Oo9426/47998 | San Diego, CA | 11-Dec-01 | Mature male | 23.94 |
| 37 | Oo9901/68407 | San Antonio, TX | 10-Jun-14 | Mature male | 14.97 |
| 38 | Oo9901/68407 | San Antonio, TX | 9-Dec-14 | Mature male | 15.46 |
| 39 | Oo8876/56411 | Orlando, FL | 11-Mar-12 | Resting female | 23.29 |
| 40 | Oo8876/56411 | Orlando, FL | 4-Dec-12 | Resting female | 24.02 |
| 41 | Oo7804/47996 | San Diego, CA | 12-Jul-07 | Resting female | 31.53 |
| 42 | Oo7804/47996 | San Diego, CA | 22-Jan-08 | Resting female | 32.06 |

Legend: MC: mother-calf pair (n = 2); SE: individual underwent stressful event (before and after, n = 2).

Table S3: Gas chromatograph (GC) column/oven conditions.

| **Temperature (°C)** | **Rate (°C/min)** | **Hold (min)** | **Total (min)** |
| --- | --- | --- | --- |
| 80.0 | 0.0 | 0.00 | 0.00 |
| 230.0 | 10.0 | 0.00 | 15.00 |
| 285.0 | 3.0 | 0.00 | 33.33 |
| 325.0 | 20.0 | 5.00 | 40.33 |

Table S4: Mass spectrometer (MS) parameters for gas chromatography (GC)-MS analysis.

| **Parameter** | **Setting** |
| --- | --- |
| MS acquisition | SIM mode |
| Ion source | EI |
| Electron impact source (EI) | 70 eV |
| Transfer line temperature | 280° C |
| Ion source temperature | 250° C |
| Acquisition delay | 8 min |
| Dwell time | 100 ms (each ion) |

SIM: selective ion monitoring

Table S5: Summary of the method for the phthalates analysis, including compounds, retention time (RT; in minutes), and ions monitored (m/z).

| **Compound name** | **Abbreviation** | **RT** | **Monitored ions**  **(m/z)** | **Monitored ions**  **(labeled standards)** |
| --- | --- | --- | --- | --- |
| Benzyl butyl phthalate | BBP | 18.736 | 91, 149, 207 | 153 |
| Dibutyl phthalate | DBP | 14.563 | 149, 227 | 153 |
| Di(2-ethylhexyl) phthalate | DEHP | 21.917 | 149, 279 | 153 |
| Diethyl phthalate | DEP | 10.667 | 149, 181, 177 | 153 |
| Dimethyl phthalate | DMP | 8.99 | 77, 137, 163 | 167 |
| Dioctyl phthalate | DOP | 25.037 | 149, 207, 279 | 153 |
| Tetrachloride-m-xylene | TCMX | 10.81 | 207 | NA |

Table S6: Results for the phthalates (PAEs) method validation, including linearity (R^2^), the method detection limit (MDL; in ng.mL^-1^), intra- and inter-day relative standard deviation (RSD), average analyte recovery (%), and matrix effect.

| **Compound** | **Linearity (R^2^)** | **MDL** | **Intra-day RSD** | **Inter-day RSD** | **Recovery (%)** | **Matrix effect** |
| --- | --- | --- | --- | --- | --- | --- |
| BBP | 0.9979 | 5.00 | 3.13 - 9.68 | 8.96 | 103 | -12.3 - 12.0 |
| DBP | 0.9983 | 5.00 | 4.46 - 26.8 | 22.6 | 115 | -31.8 - 13.8 |
| DEHP | 0.9998 | 5.00 | 13.7 - 22.1 | 18.0 | 110 | -54.4 - 94.5 |
| DEP | 0.9990 | 5.00 | 6.85 - 13.7 | 15.4 | 107 | -17.7 - 2.29 |
| DMP | 0.9988 | 5.00 | 1.50 - 6.61 | 4.36 | 104 | -6.26 - 11.2 |
| DOP | 0.9934 | 5.00 | 14.8 - 24.4 | 21.5 | 87 | -74.7 – 24.0 |

Table S7: Mean ± standard deviation, range, and detection frequency (F; %) of phthalate ester (PAEs) concentrations (ng.mL^-1^) in bottlenose dolphins by sampling location. The T-test (TT) specifies which groups were statistically significantly different (when letters are different; p < 0.05). The method detection limit (MDL) is 2.00 ng.mL^-1^.

| **PAEs** | **California**  **(N = 17)** |  |  | **Florida**  **(N = 19)** |  |  | **All**  **(N = 36)** |  |
| --- | --- | --- | --- | --- | --- | --- | --- | --- |
|  | ***Mean ± SD***  ***(range)*** | ***F*** | ***TT*** | ***Mean ± SD***  ***(range)*** | ***F*** | ***TT*** | ***Mean ± SD***  ***(range)*** | ***F*** |
| BBP | 40.10 ± 36.10  (15.20 – 93.80) | 23 | a | 85.60 ± 116.0  (12.80 – 413.0) | 68 | b | 74.90 ± 104.0  (12.80 – 413.0) | 47 |
| DBP | 83.50 ± 49.30  (31.50 – 157.0) | 29 | a | 132.0 ± 186.0  (6.000 – 492.0) | 53 | a | 116.0 ± 153.0  (6.00 – 492.0) | 42 |
| DEHP | 157.0 ± 134.0  (46.90 – 398.0) | 47 | a | 404.0 ± 376.0  (30.30 – 1251) | 63 | a | 305.0 ± 323.0  (30.30 – 1251) | 56 |
| DEP | 583.0 ± 634.0  (41.90 – 1607) | 29 | a | 269.0 ± 387.0  (5.540 – 994.0) | 47 | a | 381.0 ± 490.0  (5.540 – 1607) | 39 |
| DMP | 13.40 ± 4.080  (9.430 – 17.60) | 18 | a | 25.20 ± 26.90  (7.680 – 56.20) | 16 | a | 19.30 ± 18.40  (7.680 – 56.20) | 17 |
| DOP | <2.000 | 0 | a | 19.30 ± 6.910  (14.40 – 24.10) | 10 | a | 19.30 ± 6.910  (14.40 – 24.10) | 6 |
| ΣPAEs | 532.0 ± 575.0  (41.90 – 1739) | 24 | a | 577.0 ± 742.0  (6.000 – 2743) | 43 | a | 562.0 ± 677.0  (6.000 – 2743) | 34 |

Table S8: Mean ± standard deviation, range, and detection frequency (F; %) of phthalate ester (PAEs) concentrations (ng.mL^-1^) in killer whales by sampling location. The ANOVA test (A) specifies which groups were statistically significantly different (when letters are different in each row; p < 0.05). The method detection limit (MDL) is 2.00 ng.mL^-1^.

| **PFAS** | **California**  **(N = 21)** |  |  | **Florida**  **(N = 16)** |  |  | **Texas**  **(N = 5)** |  |  | **All**  **(N = 42)** |  |
| --- | --- | --- | --- | --- | --- | --- | --- | --- | --- | --- | --- |
|  | ***Mean ± SD***  ***(range)*** | ***F*** | ***A*** | ***Mean ± SD***  ***(range)*** | ***F*** | ***A*** | ***Mean ± SD***  ***(range)*** | ***F*** | ***A*** | ***Mean ± SD***  ***(range)*** | ***A*** |
| BBP | 172.0 ± 245.0  (7.510 – 782.0) | 71 | a | 94.00 ± 179.0  (18.10 – 652.0) | 75 | a | 700.0 ± 1071  (25.20 – 2281) | 80 | a | 210.0 ± 439.0  (7.510 – 2281) | 74 |
| DBP | 608.0 ± 1150  (8.160 – 3393) | 81 | a | 234.0 ± 333.0  (14.00 – 951.0) | 62 | a | 4128 ± 6930  (108.0 – 12130) | 60 | a | 835.0 ± 2314  (8.160 – 12130) | 71 |
| DEHP | 1497 ± 2436  (9.990 – 6729) | 76 | a | 155.0 ± 224.0  (5.370 – 843.0) | 81 | a | 17372 ± 28789 (149.0 – 60097) | 80 | a | 2892 ± 10523  (5.370 – 60097) | 79 |
| DEP | 601.0 ± 1010  (15.30 – 3589) | 76 | a | 1671 ± 3717  (56.30 – 11454) | 56 | a | 4586 ± 5870  (531.0 – 13304) | 80 | a | 1483 ± 3165  (15.30 – 13304) | 69 |
| DMP | 150.0 ± 239.0  (5.180 – 844.0) | 57 | a | 901.0 ± 1887  (7.060 – 6433) | 75 | a | 352.0 ± 448.0  (29.30 – 863.0) | 60 | a | 506.0 ± 1296  (5.180– 6433) | 64 |
| DOP | 24.00 ± NA | 5 | a | 6.560 ± 0.491  (6.220 – 6.910) | 12 | a | 13.00 ± NA | 20 | a | 12.50 ± 8.220  (6.220 – 24.00) | 9 |
| ΣPAEs | 2683 ± 4094 (42.80 –14610) | 61 | a | 2238 ± 3829 (5.370 – 13604) | 60 | a | 26021 ± 42048 (1012 – 88675) | 63 | a | 5103 ± 14880 (5.370 – 88675) | 61 |

Table S9: Mean concentration ± standard deviation, range of total phthalates (ΣPAEs; ng.mL^-1^), cortisol (ng.mL^-1^), corticosterone (ng.mL^-1^), aldosterone (pg.mL^-1^), MDA (malondialdehyde - metric for TBARS; nmol.mL^-1^) and hydrogen peroxide (HP; nmol.mL^-1^) concentrations, and sample size (N) by demographic state of bottlenose dolphins (*Tursiops truncatus*) and killer whales (*Orcinus orca*).

| **Species** | **Demographic status** | **N** | **ΣPAEs** | **Cortisol** | **Corticosterone** | **Aldosterone** | **MDA** | **HP** |
| --- | --- | --- | --- | --- | --- | --- | --- | --- |
| *Tursiops truncatus* | Calf | 6 | 691.0 ± 1168  (11.80 – 2743) | 7.890 ± 5.440  (2.120 – 16.60) | 1.410 ± 1.750  (0.100 – 4.370) | 64.40 ± 42.30  (22.00 – 118.0) | 6.940 ± 2.710  (3.870 – 10.20) | 19.80 ± 9.050  (9.780 – 32.30) |
|  | Immature male | 6 | 704.0 ± 678.0  (76.60 – 1739) | 3.800 ± 1.050  (2.400 – 4.980) | 0.350 ± 0.218  (0.030 – 0.680) | 109.0 ± 68.00  (63.40 – 243.0) | 6.160 ± 1.740  (3.130 – 7.870) | 19.60 ± 7.510  (10.40 – 28.40) |
|  | Immature female | 5 | 57.20 | 18.10 ± 17.00  (3.990 – 45.40) | 1.560 ± 1.410  (0.350 – 3.110) | 196.0 ± 279.0  (26.30 – 518.0) | 4.970 ± 1.480  (3.130 – 7.080) | 21.00 ± 10.70  (11.50 – 38.40) |
|  | Mature male | 3 | 41.90 | 6.390 ± 1.450  (4.930 – 7.820) | 0.640 ± 0.071  (0.590 – 0.690) | 0.010 | 5.060 ± 3.160  (1.820 – 8.130) | 23.40 ± 11.00  (10.90 – 31.10) |
|  | Resting female | 8 | 564.0 ± 353.0  (6.000 – 893.0) | 8.640 ± 9.140  (0.220 – 1.760) | 2.040 ± 1.630  (0.500 – 4.650) | 67.50 ± 67.20  (6.510 – 171.0) | 6.180 ± 2.820  (3.390 – 12.80) | 33.60 ± 40.10  (10.90 – 131.0) |
|  | Pregnant female | 6 | 339.0 ± 249.0  (98.90 – 731.0) | 0.738 ± 0.599  (0.220 – 1.760) | 0.212 ± 0.093  (0.130 – 0.380) | 0.010 | 8.520 ± 2.820  (4.660 – 10.70) | 45.50 ± 10.80  (25.20 – 54.50) |
|  | Lactating female | 2 | 1057 ± 1318  (125.0 – 1990) | 6.640 ± 2.470  (4.90 – 8.390) | 0.970 ± 0.721  (0.460 – 1.480) | 0.010 | 6.240 ± 1.490  (5.180 – 7.290) | 29.00 ± 11.10  (21.10 – 36.80) |
| *Orcinus orca* | Calf | 8 | 681.0 ± 398.0  (201.0 – 1253) | 7.120 ± 4.090  (0.900 – 12.70) | 0.564 ± 0.476  (0.030 – 1.060) | 57.40 ± 30.00  (25.80 – 109.0) | 67.50 ± 90.70  (5.450 – 259.0) | 315.0 ± 473.0  (34.10 – 1422) |
|  | Immature male | 5 | 786.0 ± 797.0  (223.0 – 1350) | 19.60 ± 14.00  (7.970 – 41.50) | 3.090 ± 3.170  (0.690 – 8.640) | 48.50 ± 41.60  (15.20 – 106.0) | 33.30 ± 58.00  (4.970 – 137.0) | 123.0 ± 231.0  (12.10 – 536.0) |
|  | Immature female | 6 | 2677 ± 2934  (59.50 – 7425) | 10.80 ± 3.970  (6.540 – 17.40) | 1.650 ± 0.751  (0.880 – 3.030) | 60.20 ± 19.60  (46.30 – 74.00) | 35.10 ± 35.40  (6.550 – 100.0) | 62.70 ± 93.00  (10.60 – 248.0) |
|  | Mature male | 6 | 21144 ± 33497  (1012 – 88675) | 39.20 ± 43.10  (11.20 – 124.0) | 7.260 ± 8.200  (1.150 – 23.30) | 42.60 ± 36.30  (9.350 – 94.60) | 33.10 ± 62.90  (3.920 – 161.0) | 77.30 ± 136.0  (8.670 – 351.0) |
|  | Resting female | 9 | 4165 ± 5012  (5.740 – 13604) | 23.90 ± 37.60  (2.520 – 123.0) | 5.390 ± 10.50  (0.030 – 32.70) | 44.40 ± 26.70  (18.40 – 88.60) | 22.20 ± 11.00  (7.820 – 39.10) | 54.40 ± 112.0  (10.50 – 354.0) |
|  | Pregnant female | 6 | 106.0 ± 119.0  (5.370 – 295.0) | 22.70 ± 20.20  (4.040 – 62.10) | 4.450 ± 2.480  (2.700 – 9.280) | 38.40 ± 39.30  (0.010 – 104.0) | 21.50 ± 11.60  (7.290 – 33.90) | 162.0 ± 173.0  (16.60 – 430.0) |
|  | Lactating female | 2 | 1303 ± 143.0  (1201 – 1404) | 13.40 ± 7.860  (7.800 – 18.90) | 2.180 ± 2.090  (0.710 – 3.660) | 33.50 | 19.30 ± 9.490  (12.60 – 26.00) | 196.0 ± 234.0  (30.60 – 361.0) |

Table S10: Mean ± standard deviation, range of the concentrations of the hormones cortisol (ng.mL^-1^), corticosterone (ng.mL^-1^), and aldosterone (pg.mL^-1^), and sample size (N) of delphinids by sampling location. The T-test (TT) specifies which groups were statistically significantly different (when letters are different; p < 0.05) for bottlenose dolphins (*Tursiops truncatus*). The ANOVA test (A) specifies which groups were statistically significantly different (when letters are different in each row; p < 0.05) for killer whales (*Orcinus orca*).

| **Species** | **Hormone** | **California** | ***N*** | ***TT*** | ***A*** | **Florida** | ***N*** | ***TT*** | ***A*** | **Texas** | ***N*** | ***A*** | **All** | ***N*** |
| --- | --- | --- | --- | --- | --- | --- | --- | --- | --- | --- | --- | --- | --- | --- |
| *Tursiops truncatus* | Cortisol | 9.83 ± 11.7  (2.40 – 45.4) | 17 | a | - | 5.25 ± 5.17  (0.22 – 16.6) | 19 | a | - | NA | - | - | 7.41 ± 9.01  (0.22 – 45.4) | 36 |
|  | Corticosterone | 1.05 ± 1.35  (0.03 – 4.65) | 14 | a | - | 1.02 ± 1.21  (0.10 – 4.37) | 17 | a | - | NA | - | - | 1.03 ± 1.26  (0.03 – 4.65) | 31 |
|  | Aldosterone | 122 ± 141  (0.01 – 518) | 12 | a | - | 25.1 ± 39.4  (0.01 – 118) | 15 | b | - | NA | - | - | 68.0 ± 108  (0.01 – 518) | 27 |
| *Orcinus orca* | Cortisol | 13.6 ± 10.6  (4.04 – 43.2) | 21 | - | a | 21.8 ± 30.4  (0.90 – 123) | 16 | - | a | 39.9 ± 47.5  (11.2 – 124) | 5 | a | 19.8 ± 26.1  (0.90 – 124) | 42 |
|  | Corticosterone | 2.18 ± 2.35  (0.03 – 8.64) | 21 | - | a | 4.57 ± 7.94  (0.03 – 32.7) | 16 | - | a | 6.82 ± 9.30  (1.15 –23.3) | 5 | a | 3.64 ± 6.07  (0.03 – 32.7) | 42 |
|  | Aldosterone | 43.7 ± 26.8  (15.2 – 106) | 13 | - | a | 54.5 ± 34.8  (0.01 – 109) | 12 | - | a | 40.0 ± 34.7  (9.35 – 94.6) | 5 | a | 47.4 ± 30.9  (0.01 – 109) | 30 |

Table S11: Mean ± standard deviation, range of the concentrations of the oxidative stress biomarkers MDA (malondialdehyde – metric for TBARS; nmol.mL^-1^) and hydrogen peroxide (HP; nmol.mL^-1^), and sample size (N) of delphinids by sampling location. The T-test (TT) specifies which groups were statistically significantly different (when letters are different; p < 0.05) for bottlenose dolphins (*Tursiops truncatus*). The Tukey posthoc test (T) specifies which groups were statistically significantly different (when letters are different in each row; p < 0.05) for killer whales (*Orcinus orca*).

| **Species** | **Biomarker** | **California** | ***N*** | ***TT*** | ***T*** | **Florida** | ***N*** | ***TT*** | ***T*** | **Texas** | ***N*** | ***T*** | **All** | ***N*** |
| --- | --- | --- | --- | --- | --- | --- | --- | --- | --- | --- | --- | --- | --- | --- |
| *Tursiops truncatus* | MDA | 5.35 ± 1.81  (1.82 – 8.13) | 17 | a | - | 7.40 ± 2.72  (3.87 – 12.8) | 19 | b | - | NA | - | - | 6.43 ± 2.53  (1.82 – 12.8) | 36 |
|  | HP | 20.5 ± 8.99  (10.4 – 38.4) | 17 | a | - | 34.8 ± 27.3  (9.78 – 131) | 19 | b | - | NA | - | - | 28.1 ± 21.8  (9.78 – 131) | 36 |
| *Orcinus orca* | MDA | 23.1 ± 34.0  (4.97 – 161) | 21 | - | a | 52.3 ± 66.2  (9.18 – 259) | 16 | - | b | 32.2 ± 58.5  (3.92 – 137) | 5 | ab | 35.3 ± 51.9  (3.92 – 259) | 42 |
|  | HP | 44.8 ± 71.8  (12.1 – 351) | 21 | - | a | 268 ± 348  (10.5 – 1422) | 16 | - | b | 117 ± 234  (8.67 – 536) | 5 | ab | 139 ± 252  (8.67 – 1422) | 42 |

Table S12: Linear mixed model (LMM) selection parameters of bottlenose dolphin (*Tursiops truncatus*) and killer whale (*Orcinus orca*) blood stress-related hormone (cortisol, corticosterone, and aldosterone), and oxidative stress biomarker (hydrogen peroxide – HP, and malondialdehyde - MDA) concentrations relative to month, year, location, age, demographic status, and sum of phthalates (ΣPAEs). All models used dolphin identification (ID) as a random factor to account for pseudoreplication. Models in bold represent the selected models based on the lowest Akaike Information Criterion (AIC). The degrees of freedom are indicated by DF and the fit of each model is represented by the marginal R^2^ (R^2^m; variance explained by fixed effects) and the conditional R^2^ (R^2^c; variance explained by both fixed and random effects).

| **Models** | ***Tursiops truncatus*** | | | | ***Orcinus orca*** | | | |
| --- | --- | --- | --- | --- | --- | --- | --- | --- |
|  | **DF** | **AIC** | **R^2^m** | **R^2^c** | **DF** | **AIC** | **R^2^m** | **R^2^c** |
| **1) Cortisol ~ month + year + location + age + status + ΣPAES + HP + MDA + (1\|ID)** | **36** | **85.11** | **0.43** | **1.00** | 41 | 106.5 | 0.50 | 0.50 |
| 2) Cortisol ~ month + year + location + age + status + ΣPAES + HP + (1\|ID) | 35 | 89.63 | 0.32 | 0.32 | 40 | 107.3 | 0.45 | 0.45 |
| 3) Cortisol ~ month + year + location + age + status + ΣPAES + MDA + (1\|ID) | 35 | 87.31 | 0.41 | 0.41 | 40 | 105.2 | 0.57 | 0.57 |
| 4) Cortisol ~ month + year + location + age + status + ΣPAES + (1\|ID) | 34 | 89.60 | 0.28 | 0.65 | 39 | 107.4 | 0.40 | 0.40 |
| 5) Cortisol ~ month + year + location + age + status + (1\|ID) | 33 | 87.35 | 0.34 | 0.41 | 38 | 104.3 | 0.44 | 0.44 |
| 6) Cortisol ~ month + year + location + age + (1\|ID) | 28 | 101.4 | 0.23 | 0.23 | 32 | 107.4 | 0.54 | 0.54 |
| 7) Cortisol ~ month + year + location + (1\|ID) | 27 | 99.02 | 0.23 | 0.23 | 31 | 106.1 | 0.51 | 0.51 |
| 8) Cortisol ~ month + year + age + (1\|ID) | 27 | 101.2 | 0.25 | 0.25 | 30 | 106.4 | 0.53 | 0.53 |
| 9) Cortisol ~ month + year + status + (1\|ID) | 31 | 92.92 | 0.30 | 0.30 | 35 | 107.1 | 0.44 | 0.53 |
| 10) Cortisol ~ month + year + (1\|ID) | 26 | 98.88 | 0.24 | 0.24 | 29 | 105.6 | 0.49 | 0.49 |
| 11) Cortisol ~ age + status + (1\|ID) | 10 | 88.10 | 0.49 | 0.49 | 10 | 111.9 | 0.26 | 0.26 |
| 1) Corticosterone ~ month + year + location + age + status + ΣPAES + HP + MDA + (1\|ID) | - | - | - | - | 41 | 105.8 | 0.53 | 0.53 |
| 2) Corticosterone ~ month + year + location + age + status + ΣPAES + HP + (1\|ID) | 32 | 73.48 | 0.09 | 0.40 | 40 | 106.4 | 0.48 | 0.48 |
| 3) Corticosterone ~ month + year + location + age + status + ΣPAES + MDA + (1\|ID) | 32 | 71.86 | 0.12 | 0.31 | 40 | 104.2 | 0.60 | 0.60 |
| 4) Corticosterone ~ month + year + location + age + status + ΣPAES + (1\|ID) | 31 | 72.51 | 0.14 | 0.68 | 39 | 106.0 | 0.45 | 0.45 |
| 5) Corticosterone ~ month + year + location + age + status + (1\|ID) | 30 | 69.80 | 0.22 | 0.52 | 38 | 102.5 | 0.50 | 0.50 |
| 6) Corticosterone ~ month + year + location + age + (1\|ID) | 26 | 76.51 | 0.26 | 0.32 | 32 | 106.5 | 0.53 | 0.53 |
| 7) Corticosterone ~ month + year + location + (1\|ID) | 25 | 72.95 | 0.28 | 0.28 | 31 | 105.3 | 0.50 | 0.50 |
| 8) Corticosterone ~ month + year + age + (1\|ID) | 25 | 75.86 | 0.28 | 0.28 | 30 | 103.9 | 0.55 | 0.55 |
| 9) Corticosterone ~ month + year + status + (1\|ID) | 29 | 71.27 | 0.31 | 0.31 | 35 | 103.4 | 0.55 | 0.55 |
| 10) Corticosterone ~ month + year + (1\|ID) | 24 | 71.87 | 0.31 | 0.31 | 29 | 103.8 | 0.49 | 0.49 |
| 11) Corticosterone ~ age + status + (1\|ID) | 10 | 55.10 | 0.40 | 0.42 | 10 | 104.7 | 0.32 | 0.32 |
| 1) Aldosterone ~ month + year + location + age + status + ΣPAES + HP + MDA + (1\|ID) | - | - | - | - | - | - | - | - |
| 2) Aldosterone ~ month + year + location + age + status + ΣPAES + HP + (1\|ID) | - | - | - | - | - | - | - | - |
| 3) Aldosterone ~ month + year + location + age + status + ΣPAES + MDA + (1\|ID) | - | - | - | - | - | - | - | - |
| **4) Aldosterone ~ month + year + location + age + status + ΣPAES + (1\|ID)** | - | - | - | - | **31** | **62.65** | **0.99** | **0.99** |
| 5) Aldosterone ~ month + year + location + age + status + (1\|ID) | 28 | 54.45 | 0.37 | 0.93 | 30 | 68.37 | 0.19 | 0.80 |
| 6) Aldosterone ~ month + year + location + age + (1\|ID) | 24 | 76.36 | 0.35 | 0.92 | 27 | 75.73 | 0.41 | 0.41 |
| 7) Aldosterone ~ month + year + location + (1\|ID) | 23 | 77.46 | 0.21 | 0.93 | 26 | 74.93 | 0.44 | 0.44 |
| 8) Aldosterone ~ month + year + age + (1\|ID) | 23 | 78.58 | 0.38 | 0.89 | 25 | 75.87 | 0.44 | 0.44 |
| 9) Aldosterone ~ month + year + status + (1\|ID) | 27 | 63.03 | 0.73 | 0.87 | 29 | 68.57 | 0.53 | 0.53 |
| 10) Aldosterone ~ month + year + (1\|ID) | 22 | 80.36 | 0.12 | 0.93 | 24 | 75.54 | 0.43 | 0.43 |
| 11) Aldosterone ~ age + status + (1\|ID) | 10 | 72.39 | 0.67 | 0.93 | 10 | 90.42 | 0.18 | 0.18 |
| 1) HP ~ month + year + location + age + status + ΣPAES + cortisol + MDA + (1\|ID) | 36 | 82.91 | 0.78 | 0.78 | 41 | 106.3 | 0.76 | 0.76 |
| 2) HP ~ month + year + location + age + status + ΣPAES + cortisol + (1\|ID) | 35 | 88.09 | 0.30 | 0.30 | 40 | 101.1 | 0.39 | 1.00 |
| 3) HP ~ month + year + location + age + status + ΣPAES + corticosterone + MDA + (1\|ID) | - | - | - | - | 41 | 106.1 | 0.75 | 0.75 |
| 4) HP ~ month + year + location + age + status + ΣPAES + corticosterone + (1\|ID) | 32 | 73.48 | 0.09 | 0.09 | 40 | 100.9 | 0.39 | 1.00 |
| 5) HP ~ month + year + location + age + status + ΣPAES + aldosterone + MDA + (1\|ID) | - | - | - | - | - | - | - | - |
| 6) HP ~ month + year + location + age + status + ΣPAES + aldosterone + (1\|ID) | - | - | - | - | - | - | - | - |
| 7) HP ~ month + year + location + age + status + ΣPAES + MDA + (1\|ID) | 35 | 80.15 | 0.78 | 0.78 | 40 | 102.2 | 0.41 | 1.00 |
| 8) HP ~ month + year + location + age + status + ΣPAES + (1\|ID) | 34 | 86.71 | 0.29 | 0.29 | 39 | 95.13 | 0.41 | 1.00 |
| 9) HP ~ month + year + location + age + status + MDA + (1\|ID) | 34 | 75.91 | 0.78 | 0.78 | 39 | 95.81 | 0.41 | 1.00 |
| **10) HP ~ month + year + location + age + status + (1\|ID)** | 33 | 83.50 | 0.33 | 0.33 | **38** | **90.53** | **0.42** | **1.00** |
| 11) HP ~ month + year + location + age + (1\|ID) | 28 | 88.27 | 0.37 | 0.50 | 32 | 109.1 | 0.63 | 0.88 |
| 12) HP ~ month + year + location + (1\|ID) | 27 | 84.74 | 0.37 | 0.51 | 31 | 108.8 | 0.40 | 0.99 |
| 13) HP ~ month + year + age + (1\|ID) | 27 | 87.14 | 0.38 | 0.49 | 30 | 112.2 | 0.65 | 0.74 |
| 14) HP ~ month + year + status + (1\|ID) | 31 | 86.52 | 0.30 | 0.51 | 35 | 105.9 | 0.76 | 0.76 |
| 15) HP ~ month + year + age + status + (1\|ID) | 32 | 84.73 | 0.37 | 0.37 | 36 | 97.29 | 0.34 | 1.00 |
| 16) HP ~ month + year + (1\|ID) | 26 | 83.65 | 0.37 | 0.51 | 29 | 111.4 | 0.65 | 0.71 |
| 17) HP ~ age + status + (1\|ID) | 10 | 74.10 | 0.23 | 0.37 | 10 | 129.0 | 0.17 | 0.80 |
| 1) MDA ~ month + year + location + age + status + ΣPAES + cortisol + HP + (1\|ID) | 36 | 81.20 | 0.87 | 0.87 | 41 | 104.6 | 0.80 | 0.80 |
| 2) MDA ~ month + year + location + age + status + ΣPAES + cortisol + (1\|ID) | 35 | 82.95 | 0.50 | 0.50 | 40 | 103.0 | 0.81 | 0.81 |
| 3) MDA ~ month + year + location + age + status + ΣPAES + corticosterone + HP + (1\|ID) | - | - | - | - | 41 | 104.3 | 0.80 | 0.80 |
| 4) MDA ~ month + year + location + age + status + ΣPAES + corticosterone + (1\|ID) | 32 | 71.86 | 0.11 | 0.44 | 40 | 102.9 | 0.80 | 0.80 |
| 5) MDA ~ month + year + location + age + status + ΣPAES + aldosterone + HP + (1\|ID) | - | - | - | - | - | - | - | - |
| 6) MDA ~ month + year + location + age + status + ΣPAES + aldosterone + (1\|ID) | - | - | - | - | - | - | - | - |
| 7) MDA ~ month + year + location + age + status + ΣPAES + HP + (1\|ID) | 35 | 77.32 | 0.84 | 0.84 | 40 | 103.6 | 0.77 | 0.77 |
| 8) MDA ~ month + year + location + age + status + ΣPAES + (1\|ID) | 34 | 81.07 | 0.41 | 0.41 | 39 | 103.8 | 0.70 | 0.70 |
| 9) MDA ~ month + year + location + age + status + HP + (1\|ID) | 34 | 71.57 | 0.85 | 0.85 | 39 | 99.68 | 0.80 | 0.80 |
| 10) MDA ~ month + year + location + age + status + (1\|ID) | 33 | 76.27 | 0.47 | 0.47 | 38 | 100.5 | 0.63 | 0.78 |
| 11) MDA ~ month + year + location + age + (1\|ID) | 28 | 75.34 | 0.49 | 0.49 | 32 | 106.7 | 0.67 | 0.67 |
| 12) MDA ~ month + year + location + (1\|ID) | 27 | 70.48 | 0.50 | 0.50 | 31 | 107.9 | 0.58 | 0.62 |
| 13) MDA ~ month + year + age + (1\|ID) | 27 | 76.66 | 0.33 | 0.66 | 30 | 110.2 | 0.57 | 0.57 |
| 14) MDA ~ month + year + status + (1\|ID) | 31 | 78.96 | 0.32 | 0.53 | 35 | 108.5 | 0.54 | 0.54 |
| 15) MDA ~ month + year + age + status + (1\|ID) | 32 | 78.50 | 0.35 | 0.52 | 36 | 108.5 | 0.51 | 0.51 |
| 16) MDA ~ month + year + (1\|ID) | 26 | 72.43 | 0.36 | 0.60 | 29 | 108.7 | 0.55 | 0.55 |
| 17) MDA ~ age + status + (1\|ID) | 10 | 50.01 | 0.16 | 0.43 | 10 | 126.6 | 0.16 | 0.60 |
|  |  |  |  |  |  |  |  |  |
|  |  |  |  |  |  |  |  |  |
|  |  |  |  |  |  |  |  |  |
|  |  |  |  |  |  |  |  |  |
|  |  |  |  |  |  |  |  |  |
|  |  |  |  |  |  |  |  |  |
|  |  |  |  |  |  |  |  |  |
|  |  |  |  |  |  |  |  |  |
|  |  |  |  |  |  |  |  |  |
|  |  |  |  |  |  |  |  |  |

Table S13: Parameter estimates from the statistically significant selected linear mixed models for bottlenose dolphin (*Tursiops truncatus*; cortisol model 1) and killer whale (*Orcinus orca*; aldosterone model 4 and hydrogen peroxide model 10) serum concentrations. Asterisks indicate significance at the 0.05 (*), 0.01 (**), and 0.001 (***) levels.

| **Model** | **Random effects** | **Variance** | **Standard Deviation** | **Fixed Effects** | **Estimate** | **Standard Error** | **p value** |
| --- | --- | --- | --- | --- | --- | --- | --- |
| **Cortisol** | Dolphin ID | 1.4576208 | 1.207320 | Month (January) | -5.15727 | 1.87511 | 0.110677 |
| **Model 1** | Residuals | 0.0000357 | 0.005975 | Month (February) | -3.24382 | 1.83614 | 0.219323 |
| ***T. truncatus*** |  |  |  | Month (March) | 3.05949 | 0.94589 | 0.083750 |
|  |  |  |  | Month (April) | -2.95738 | 2.99156 | 0.427073 |
|  |  |  |  | Month (May) | 1.91673 | 0.19139 | 0.009824 ** |
|  |  |  |  | Month (June) | -3.44779 | 3.84333 | 0.464345 |
|  |  |  |  | Month (July) | -5.99495 | 1.81746 | 0.080912 |
|  |  |  |  | Month (August) | 2.79038 | 0.38345 | 0.018365 * |
|  |  |  |  | Month (September) | 3.63484 | 0.96548 | 0.063868 |
|  |  |  |  | Month (October) | 9.61189 | 3.5203 | 0.112039 |
|  |  |  |  | Month (November) | 1.5265 | 0.02911 | 0.000364 *** |
|  |  |  |  | Month (December) | -2.32607 | 2.1736 | 0.396582 |
|  |  |  |  | Year (2005) | 4.12849 | 2.44526 | 0.233398 |
|  |  |  |  | Year (2008) | 4.1509 | 2.99338 | 0.299875 |
|  |  |  |  | Year (20010) | 3.61756 | 3.3487 | 0.392965 |
|  |  |  |  | Year (20011) | -13.18148 | 7.46323 | 0.219404 |
|  |  |  |  | Year (2012) | -0.89182 | 3.28735 | 0.811605 |
|  |  |  |  | Year (2013) | -9.27034 | 6.63328 | 0.297096 |
|  |  |  |  | Year (2014) | -8.48428 | 6.72207 | 0.334142 |
|  |  |  |  | Year (2015) | -10.08037 | 5.46455 | 0.206385 |
|  |  |  |  | Year (2016) | -7.13532 | 5.75299 | 0.34064 |
|  |  |  |  | Year (2017) | -11.80931 | 4.95653 | 0.140075 |
|  |  |  |  | Year (2018) | -2.83881 | 6.06151 | 0.685628 |
|  |  |  |  | Year (2020) | -16.18786 | 6.29721 | 0.123836 |
|  |  |  |  | Location (Florida) | 13.26486 | 3.28454 | 0.056194 |
|  |  |  |  | Age | 2.97427 | 1.54256 | 0.193644 |
|  |  |  |  | Status (Immature female) | 14.29014 | 6.28269 | 0.150768 |
|  |  |  |  | Status (Immature male) | 1.89442 | 1.60128 | 0.358358 |
|  |  |  |  | Status (Lactating female) | -10.1046 | 4.873 | 0.173847 |
|  |  |  |  | Status (Pregnant female) | -8.1648 | 4.73333 | 0.226677 |
|  |  |  |  | Status (Resting female) | 0.28335 | 5.15389 | 0.961154 |
|  |  |  |  | HP | 2.63647 | 0.37251 | 0.019385 * |
|  |  |  |  | MDA | -6.15506 | 1.28033 | 0.040649 * |
|  |  |  |  | ΣPAES | 0.04452 | 0.22482 | 0.861334 |
| **Aldosterone** | Dolphin ID | 0.007653 | 0.08748 | Month (January) | 10.78671 | 0.72043 | 0.0425 * |
| **Model 4** | Residuals | 0.004838 | 0.06956 | Month (February) | -3.20542 | 0.24091 | 0.0478 * |
| ***O. orca*** |  |  |  | Month (March) | -5.01974 | 0.2767 | 0.0351 * |
|  |  |  |  | Month (April) | 12.34069 | 0.37011 | 0.0191 * |
|  |  |  |  | Month (May) | -14.70342 | 0.6402 | 0.0277 * |
|  |  |  |  | Month (June) | 3.61456 | 0.29558 | 0.0519 |
|  |  |  |  | Month (July) | 18.00109 | 0.8712 | 0.0308 * |
|  |  |  |  | Month (September) | -12.10879 | 0.44997 | 0.0236 * |
|  |  |  |  | Month (October) | -5.6443 | 0.33397 | 0.0376 * |
|  |  |  |  | Month (November) | -9.24452 | 0.46963 | 0.0323 * |
|  |  |  |  | Month (December) | 5.7467 | 0.3966 | 0.0439 * |
|  |  |  |  | Year (1997) | -15.5248 | 0.9901 | 0.0405 * |
|  |  |  |  | Year (2001) | 12.48471 | 0.53567 | 0.0273 * |
|  |  |  |  | Year (2005) | 5.44882 | 0.41218 | 0.0481 * |
|  |  |  |  | Year (2006) | -5.77285 | 0.51058 | 0.0562 |
|  |  |  |  | Year (2008) | 6.80026 | 0.32303 | 0.0302 * |
|  |  |  |  | Year (2009) | 6.5167 | 0.31143 | 0.0304 * |
|  |  |  |  | Year (2012) | -9.17269 | 0.5676 | 0.0393 * |
|  |  |  |  | Year (2013) | -0.54748 | 0.27355 | 0.295 |
|  |  |  |  | Year (2014) | -0.42764 | 0.26231 | 0.3503 |
|  |  |  |  | Year (2015) | 8.85315 | 0.46559 | 0.0334 * |
|  |  |  |  | Year (2016) | 16.50276 | 0.70292 | 0.0271 * |
|  |  |  |  | Location (Florida) | 11.17977 | 0.54496 | 0.0310 * |
|  |  |  |  | Location (Texas) | 11.35159 | 0.56994 | 0.0319 * |
|  |  |  |  | Age | -4.57414 | 0.20268 | 0.0282 * |
|  |  |  |  | Status (Immature male) | 7.91414 | 0.47864 | 0.0385 * |
|  |  |  |  | Status (Lactating female) | 22.28849 | 0.96357 | 0.0275 * |
|  |  |  |  | Status (Pregnant female) | 3.38281 | 0.36513 | 0.0685 |
|  |  |  |  | ΣPAES | -1.21678 | 0.06488 | 0.0339 * |
| **Hydrogen Peroxide** | Dolphin ID | 2.268849 | 1.50627 | Month (January) | 1.38931 | 1.37567 | 0.36831 |
| **Model 10** | Residuals | 0.001031 | 0.03211 | Month (February) | 2.20011 | 0.20651 | 0.00273 ** |
| ***O. orca*** |  |  |  | Month (March) | 1.79028 | 0.24122 | 0.00778 ** |
|  |  |  |  | Month (April) | 3.83996 | 1.96961 | 0.12032 |
|  |  |  |  | Month (May) | 0.62442 | 0.14665 | 0.01489 * |
|  |  |  |  | Month (June) | 1.86898 | 0.21421 | 0.00402 ** |
|  |  |  |  | Month (July) | 0.78084 | 0.19288 | 0.05108 |
|  |  |  |  | Month (August) | 0.61421 | 0.23809 | 0.08536 |
|  |  |  |  | Month (September) | 2.5587 | 0.25995 | 0.00519 ** |
|  |  |  |  | Month (October) | 1.23128 | 0.22021 | 0.00825 ** |
|  |  |  |  | Month (November) | 0.70932 | 1.36478 | 0.62998 |
|  |  |  |  | Month (December) | 2.34921 | 0.23813 | 0.00197 ** |
|  |  |  |  | Year (1997) | -0.46736 | 0.51381 | 0.40936 |
|  |  |  |  | Year (2000) | 0.83383 | 0.76341 | 0.3325 |
|  |  |  |  | Year (2001) | 6.16351 | 3.4395 | 0.1475 |
|  |  |  |  | Year (2005) | -1.03047 | 1.4192 | 0.50753 |
|  |  |  |  | Year (2006) | 0.35911 | 1.42617 | 0.81332 |
|  |  |  |  | Year (2007) | -0.29969 | 2.84485 | 0.92112 |
|  |  |  |  | Year (2008) | -0.51781 | 1.74974 | 0.78152 |
|  |  |  |  | Year (2009) | -3.17193 | 1.93564 | 0.17584 |
|  |  |  |  | Year (2010) | -0.16363 | 1.5934 | 0.92309 |
|  |  |  |  | Year (2012) | -0.59816 | 1.75894 | 0.7507 |
|  |  |  |  | Year (2013) | -1.1766 | 1.9018 | 0.56935 |
|  |  |  |  | Year (2014) | -0.61493 | 1.94261 | 0.76721 |
|  |  |  |  | Year (2015) | -0.24768 | 1.81368 | 0.8979 |
|  |  |  |  | Year (2016) | -2.08775 | 2.02805 | 0.36138 |
|  |  |  |  | Year (2017) | -1.81428 | 2.20305 | 0.45613 |
|  |  |  |  | Location (Florida) | 1.05159 | 1.0807 | 0.38542 |
|  |  |  |  | Location (Texas) | 3.70787 | 1.40285 | 0.05688 |
|  |  |  |  | Age | 0.02336 | 0.71353 | 0.97542 |
|  |  |  |  | Status (Immature female) | -1.18414 | 2.0205 | 0.58879 |
|  |  |  |  | Status (Immature male) | -2.93426 | 2.22858 | 0.25784 |
|  |  |  |  | Status (Lactating female) | -3.25894 | 2.32341 | 0.23281 |
|  |  |  |  | Status (Pregnant female) | -2.72408 | 2.31034 | 0.30319 |
|  |  |  |  | Status (Resting female) | -1.5292 | 2.30095 | 0.54221 |
|  |  |  |  | Status (Mature male) | -6.56394 | 2.81331 | 0.07874 |


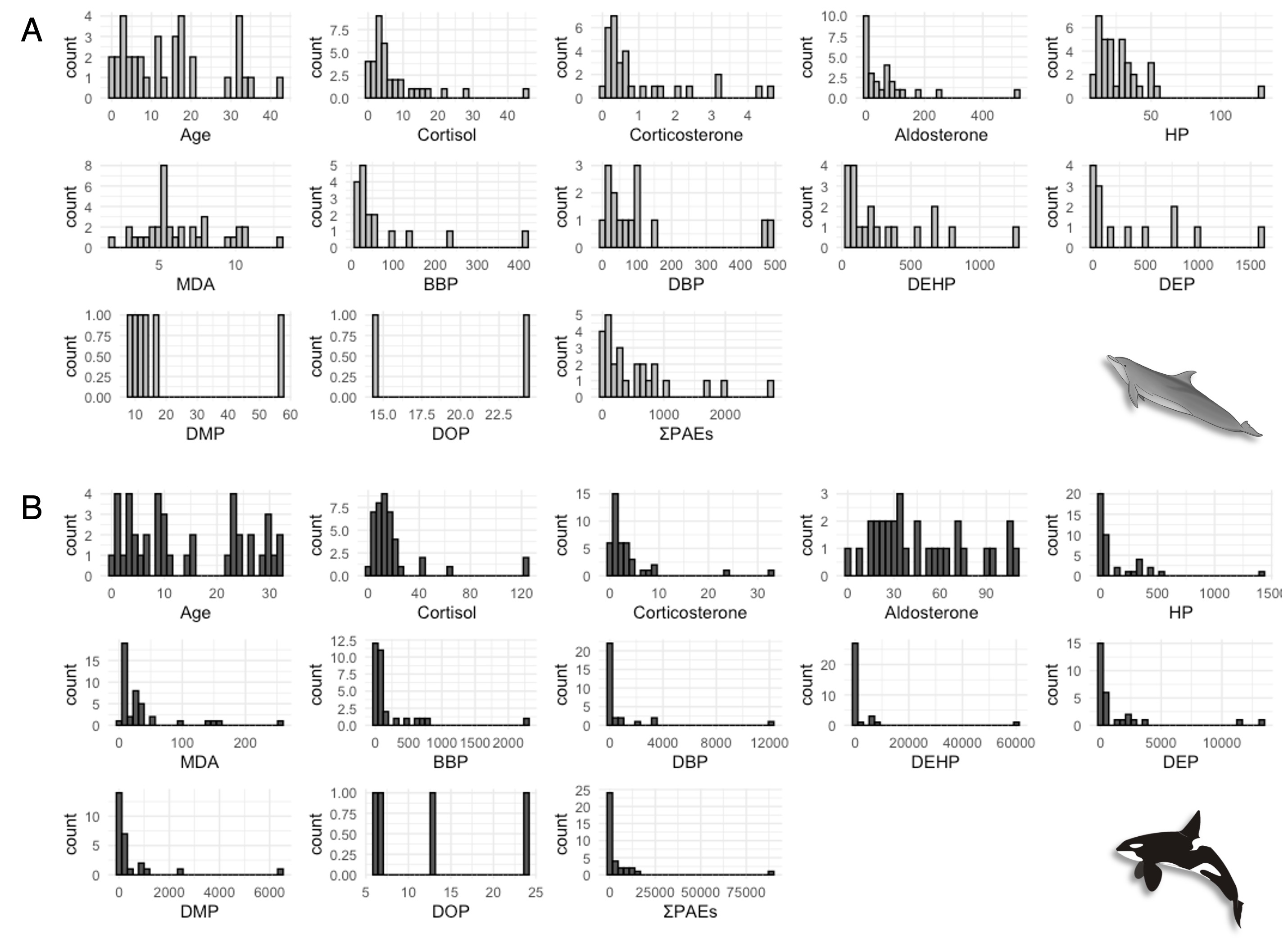


Figure S1: Distribution of the numeric variables analyzed from serum samples of bottlenose dolphins (A) and killer whales (B) from three SeaWorld facilities (California, Florida, and Texas), including age, hormones, oxidative stress biomarkers, 6 compounds of PAEs and the sum of PAEs (ΣPAEs).

**
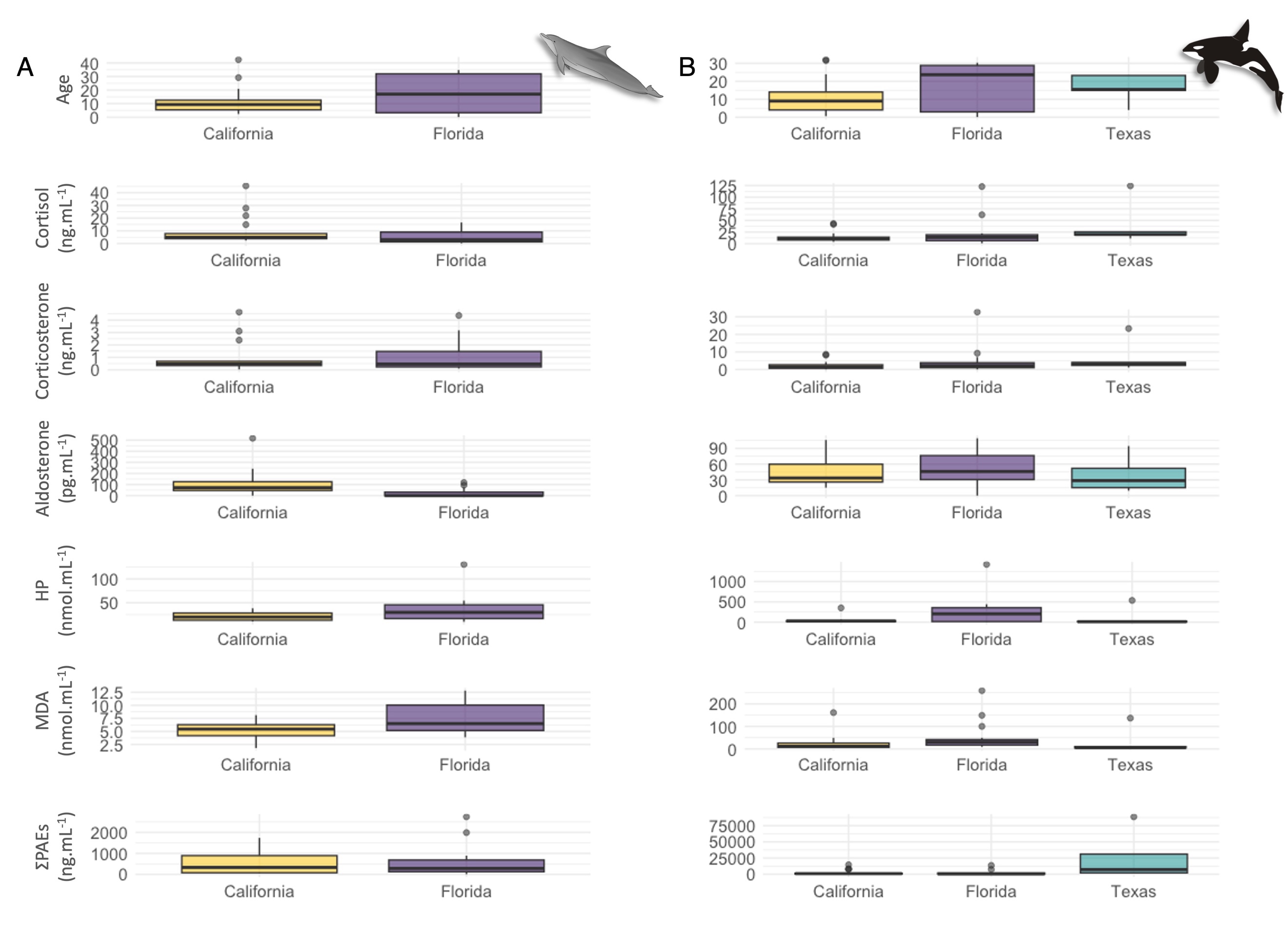
**

Figure S2: Boxplots of age, hormones (cortisol, corticosterone, and aldosterone), and oxidative stress biomarkers (HP and MDA) in bottlenose dolphins (A) and killer whales (B) by SeaWorld facilities (California, Florida, and Texas). Bottlenose dolphins were only sampled in two facilities (California and Florida). In the boxplots, the limit of the box closest to zero indicates the 25^th^ percentile, the black line within the box indicates the median and the limit of the box farthest from zero indicates the 75^th^ percentile. The whiskers represent extreme observations, and the gray dots represent the outliers. Individual dolphins may be represented multiple times in these plots as they were re-sampled over time.

**
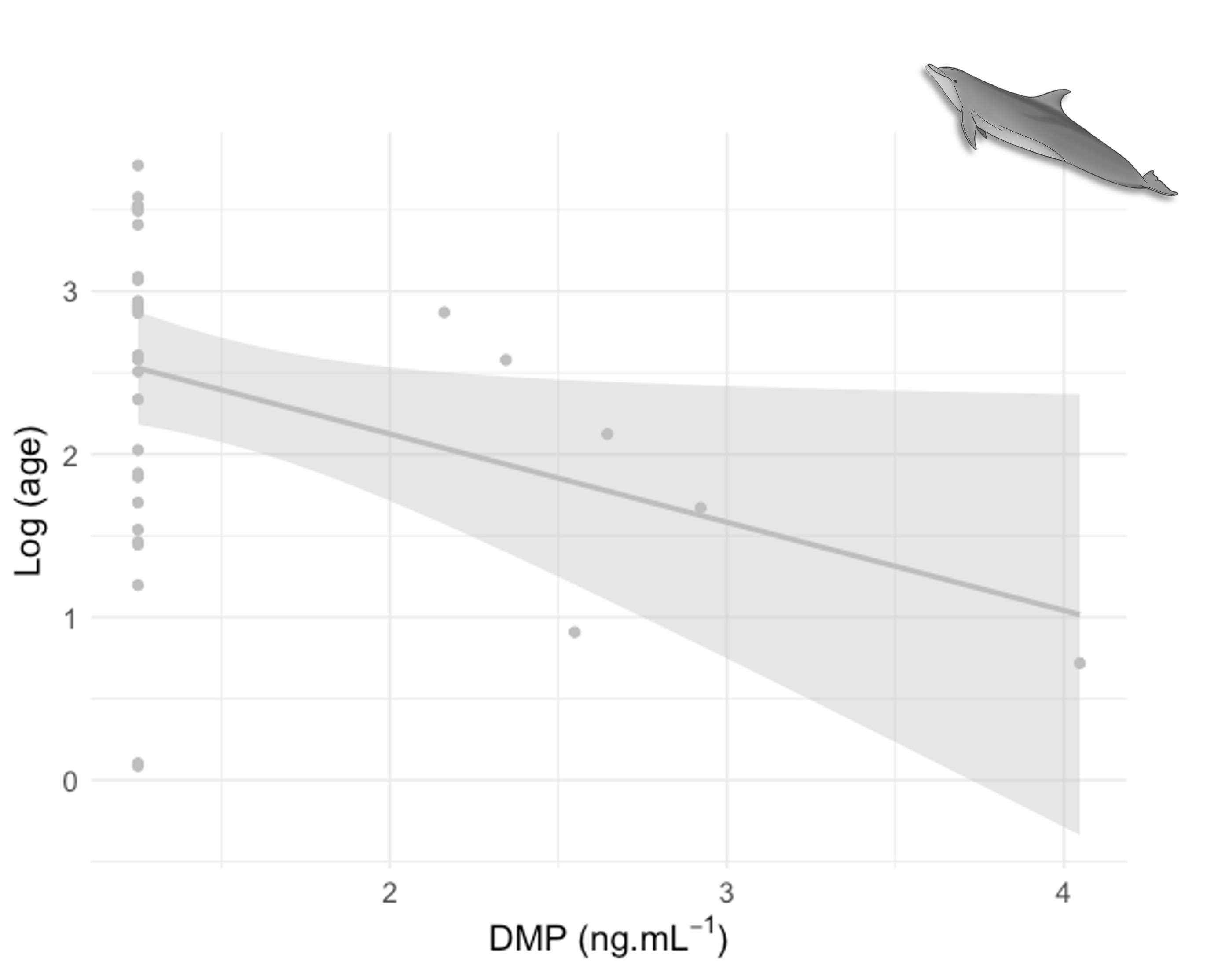
**

Figure S3: Significant linear correlation between age (log + 1) and DMP (log + 1) in bottlenose dolphins. Individual dolphins may be represented multiple times in these plots as some were re-sampled over the years.


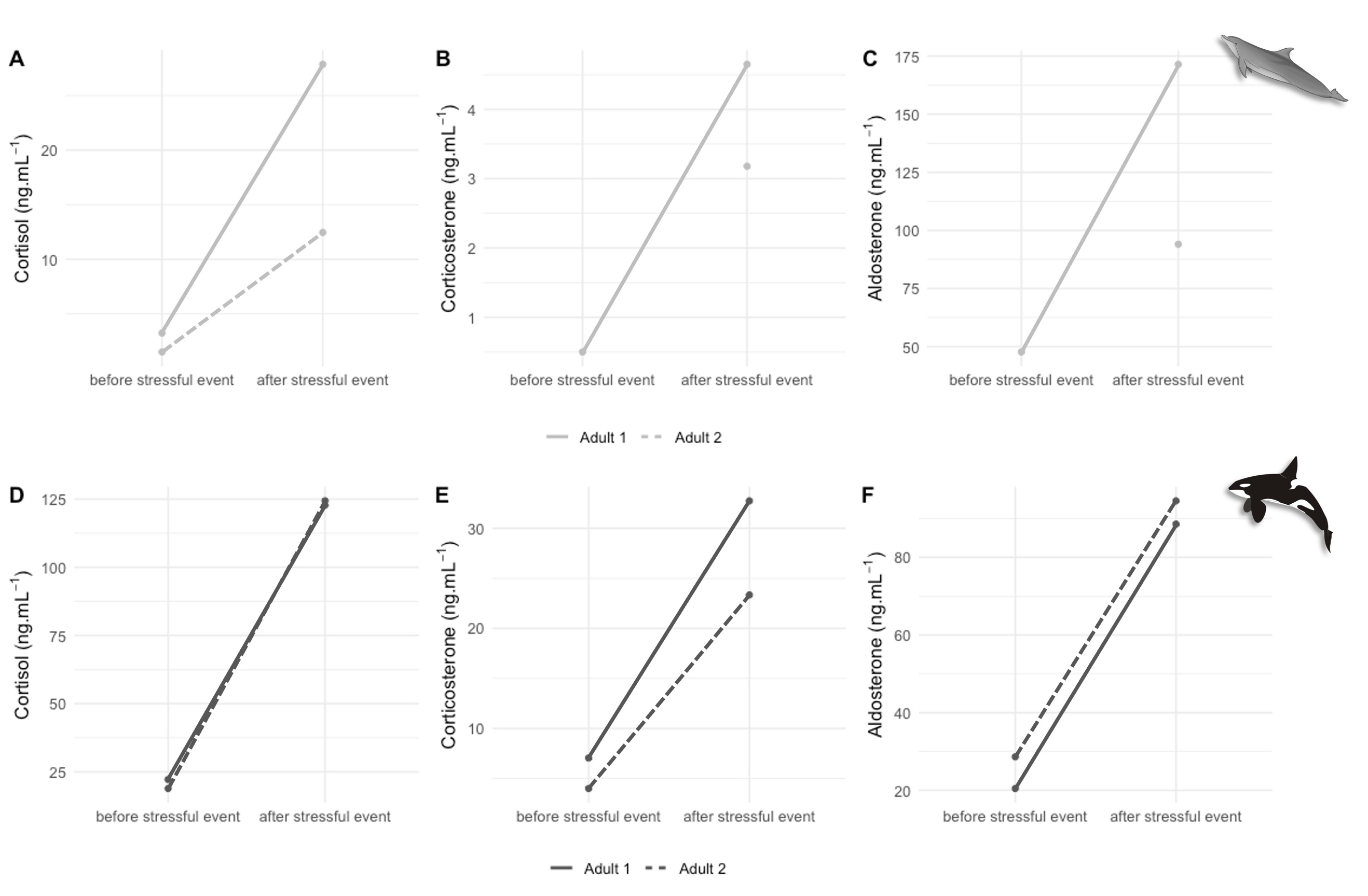


Figure S4: Temporal variation of the hormones cortisol (A and D), corticosterone (B and E), and aldosterone (C and F) in mature individuals of bottlenose dolphins (n = 2) and killer whales (n = 2) before and after being raised on a lifting floor or out of the water for a medical procedure (i.e., stressful event). Due to the low sample volume, no information on corticosterone and aldosterone was available for bottlenose dolphin adult 2 before the stressful event.


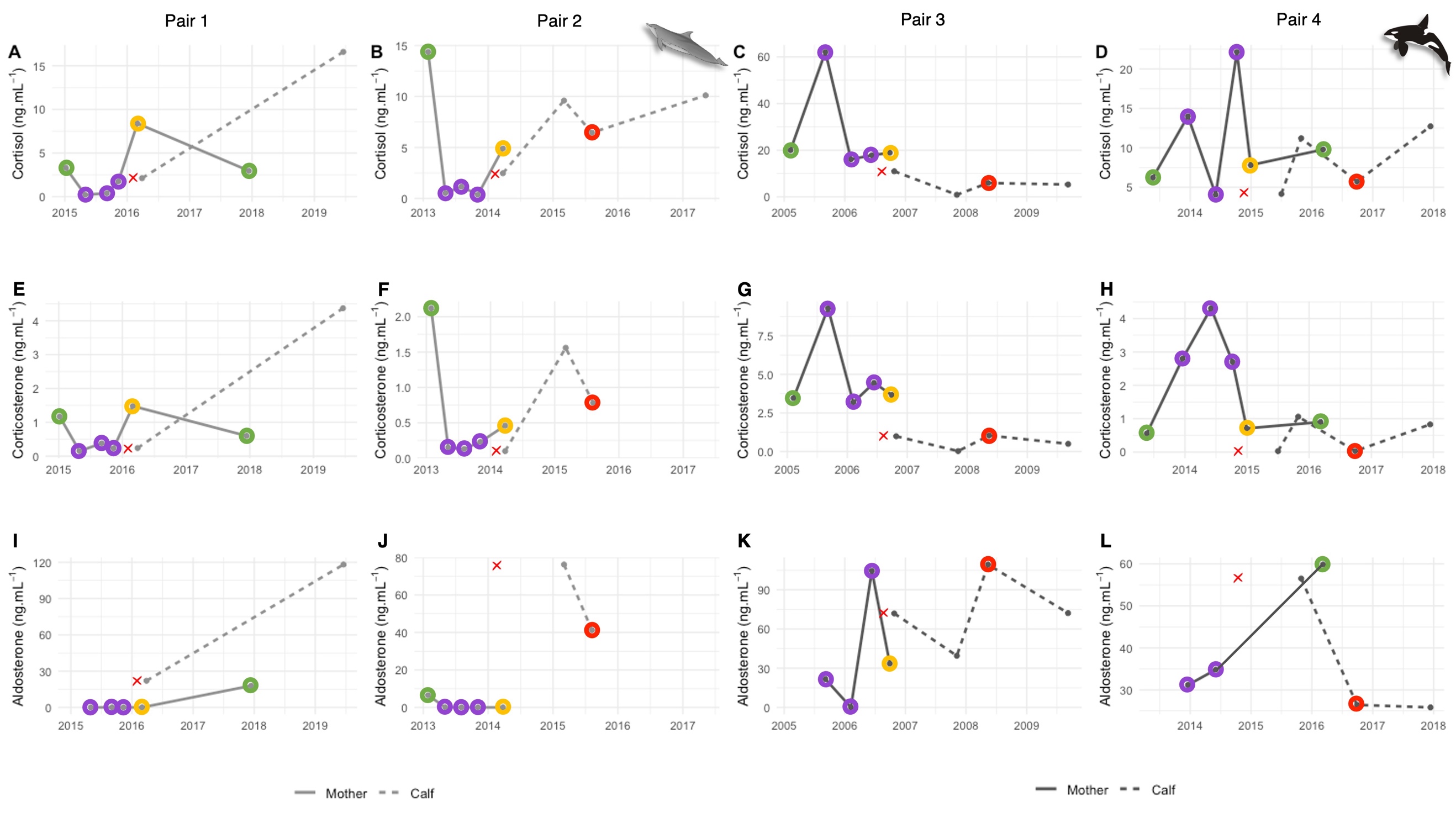
Figure S5: Temporal variation of steroid hormones (cortisol, corticosterone, and aldosterone) in mother-calf pairs of bottlenose dolphins (A, B, E, F, I, and J) and killer whales (C, D, G, H, K, and L). Demographic statuses are color-coded: green circles = females were resting (not pregnant or lactating); purple circles = females were pregnant; yellow circles = females were lactating; and red circles = calves weaned. Red xes indicate parturition dates (A, E, I: 23-Feb-2016; B, F, J: 12-Feb-2014; C, G, K: 18-Sep-2006; and D, H, L: 2-Dec-2014).


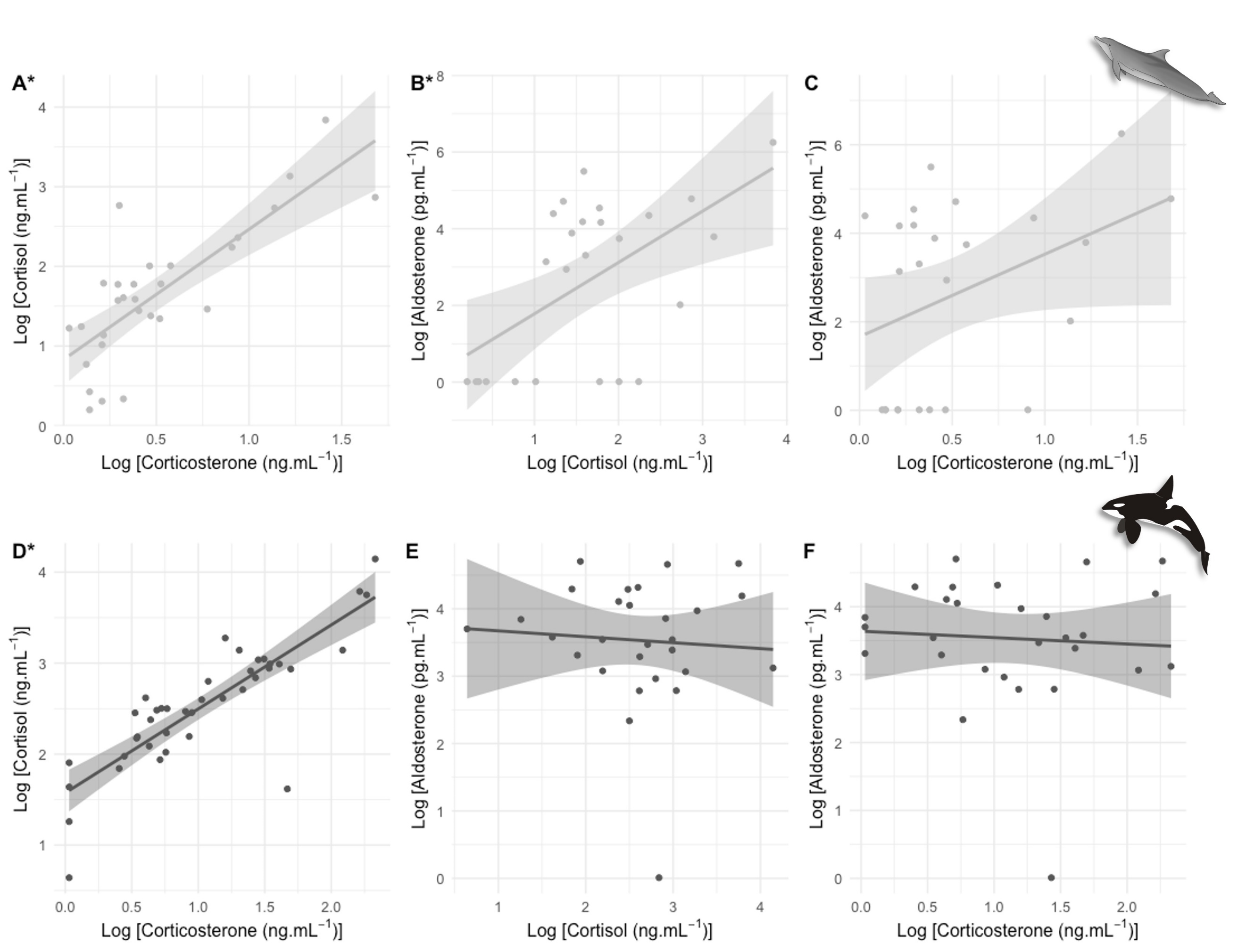


Figure S6: Linear correlations between steroid hormones (cortisol, corticosterone, and aldosterone; log+1) in bottlenose dolphins (A, B, and C) and killer whales (D, E, and F). Individual dolphins may be represented multiple times in these plots as some were re-sampled over the years. Individuals undergoing known stressful events were excluded from this analysis. Asterisks indicate significance at the 0.05 level.


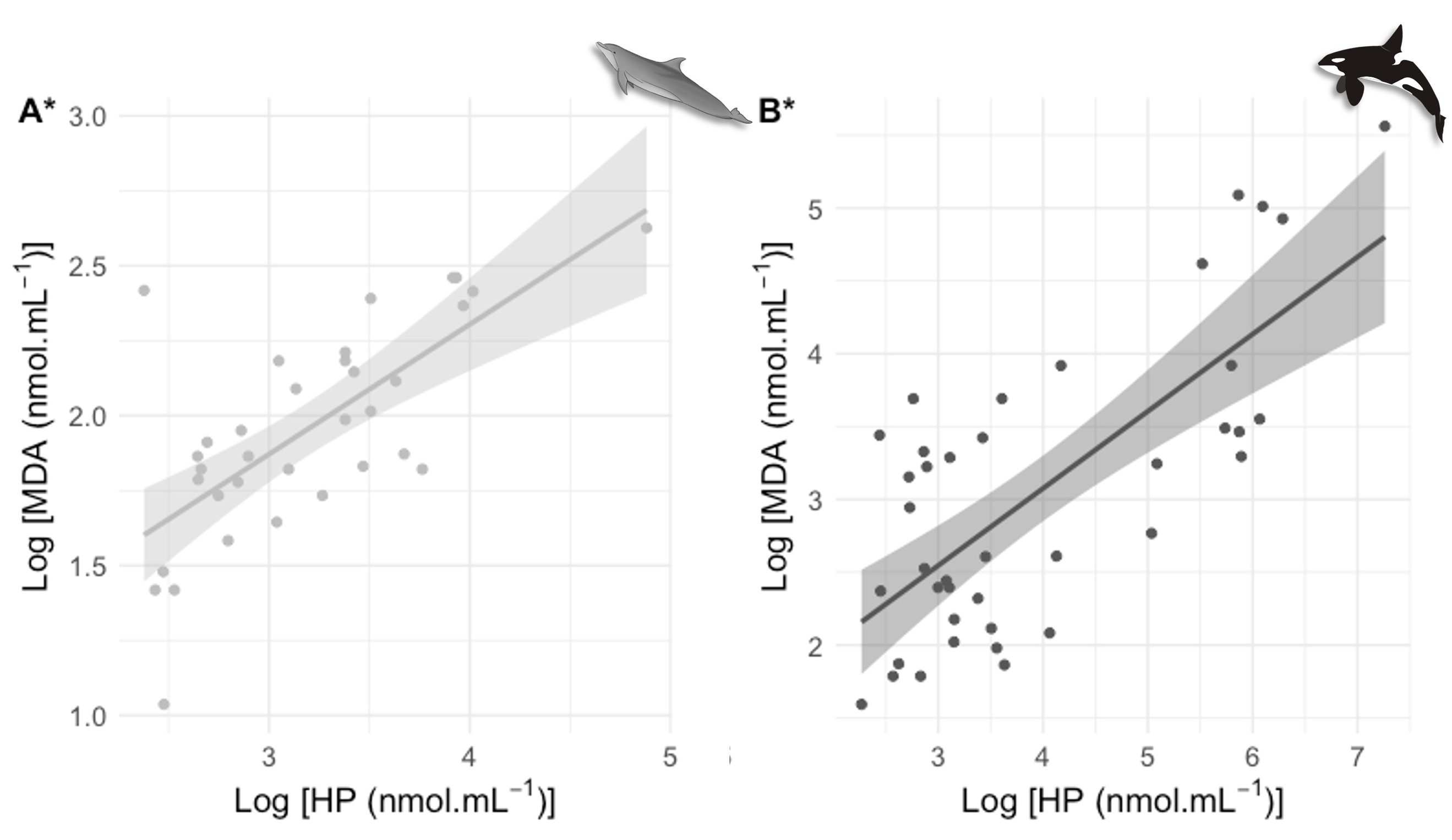


Figure S7: Linear correlations between the oxidative stress biomarkers hydrogen peroxide (HP) and malondialdehyde (MDA; log+1) in bottlenose dolphins (A) and killer whales (B). Individual dolphins may be represented multiple times in these plots as some were re-sampled over the years. Asterisks indicate significance at the 0.05 level.
